# Cochlear implant re-mapping informed by measures of viability of the electrode-neural interface: a systematic review with meta-analysis

**DOI:** 10.1038/s41598-025-09610-x

**Published:** 2025-07-30

**Authors:** Jason Tzu-Hsien Lien, Ben Williges, Deborah Vickers

**Affiliations:** 1 SOUND Lab, Department of Clinical Neurosciences, University of Cambridge, Cambridge, UK; 2 ENT-Department, University Hospital Essen, Essen, Germany

**Keywords:** Translational research, Medical research, Outcomes research, Auditory system, Biomedical engineering

## Abstract

The electrode to auditory nerve interface (ENI) is often considered a bottleneck for information transmission for listeners using a cochlear implant (CI). Clinically, it could be beneficial to have a CI programming plan based on optimising information flow based on an individual’s ENI status. This review explores whether re-mappings informed by the viability of ENI can improve the speech perception (noise and/or quiet) of adult CI users. Six databases (MEDLINE, EMBASE, TRIP, Scopus, Web of Science, CINAHL), were searched in April 2024 to identify studies that compared an experimental CI mapping method informed by an ENI measure with the routine clinical mapping among adult CI users. A customised questionnaire was created modified from established critical appraisal tools to assess the risk of bias. Data was extracted to compute a standardised mean difference between the control and experimental maps (Cohen’s d) and its variance for each article. A mixed-effect model was used to estimate the combined Cohen’s d. Linear Regressions were used to probe potential interactions. Thirty articles, mostly within-subject map crossover studies and one RCT, were included. Re-mappings informed by ENI yielded a moderate and significant effect size of 0.48 on speech-in-noise perception. Looking into subgroups, site selection interventions yielded a moderate and significant (*p* = 0.005) effect size of 0.59. Some site selection interventions were particularly successful while being informed by the low-rate threshold, modulation detection threshold, and electrode discrimination, yielding large and significant effect sizes around 1–1.5. Interventions aiming to reduce the Frequency-to-Place Mismatch by altering the frequency allocation yielded an insignificant (*p* = 0.32) effect size of 0.47 due to the large variability between and within studies. The variability of outcomes remains substantial both within and between studies. The same intervention is often conducted by the same research group and hence replications at different labs could further strengthen the result. Based on the synthesised result, re-mappings informed by ENI measure could provide better CI hearing to individuals.

## Introduction

Cochlear implants (CIs) are one of the most successful medical devices helping well over a million recipients across the globe^1,2^. CIs are the intervention of choice for many severe-to-profoundly deaf children and adults who do not receive adequate benefit from hearing aids. Most individuals with CIs achieve high levels of speech understanding in quiet (often 100% scores for sentences) and hear better than using hearing aids alone^3–6^. Despite the great success, there is still a large variability in outcomes, which is affected by many factors such as age of implantation, years of hearing deprivation, or the listening experience with the device^2,7–10^. Many researchers suggest that the viability of the electrode-neural interface (ENI, the interface between the CI electrode array and the auditory nerve) is one of the most important factors underpinning CI performance and it needs to be better understood^11–13^. Modelling work has shown that the largest loss of information transmission happens at the ENI^14^. Potentially by accurately assessing an individual’s ENI status, a personalised intervention plan aimed at delivering better performance could be formed. This systematic review initially outlines the main ENI measures for informing CI re-mapping approaches and evaluates research where these experimental ENI interventions are compared to standard care. Subsequently, we conducted a meta-analysis to estimate the clinical efficacy of such interventions.

### Electrode-neural interface

In CI hearing, the electrode array is typically inserted into the scala tympani to replace the role of the inner hair cells (IHC). Instead of the trans-synapse interaction initiated by the neural transmitters, a pair of electrodes is activated to drive local potential changes and hence currents initiating the action potentials along the dendrites of the stimulated spiral ganglion cells (SGC). This crucial interface between the CI electrode and the auditory nerve is often termed the Electrode-Neural Interface (ENI).

**Fig. 1:**
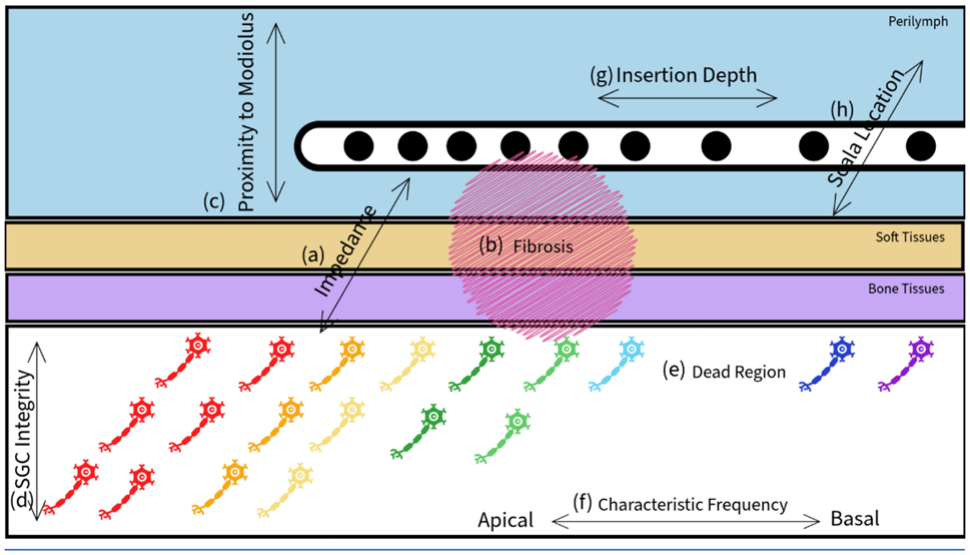
Common topics explored that affect the quality of the electrode-neural interface. The four sections marked by the rectangles, from top to down, represent the electrode array in the Perilymph, the soft tissues, the bone tissues, and the SGCs in the modiolus, respectively. Each filled circle represents an electrode contact of the array. Each neurone icon represents a population of SGCs. The colour of each icon marks the CF of the population. (**a**) The electrical impedance is determined by the resistance of the path between the active and ground electrode passing through the stimulated SGCs. (**b**) Fibrous tissue and newly formed bony tissue, which are much less conductive, occupy space in cochlea instead of the conductive fluids increases electrical resistance. (**c**) Top represents further away from the modiolus. (**d**) Layer of SGCs represent the integrity of them. (**e**) A frequency range without any functioning SGCs represents a dead region. (**f**) Left side represents the apical side and lower CF. (**g**) Left side represents a deeper insertion depth. (**h**) Scala location (scala-media, scala-tympani or scala-vestibuli) of the electrode array. Note that scala-media is filled with Endolympth not Perilymph.

To represent a sound, a conventional CI mainly performs two essential tasks per timeframe, coding the frequency and intensity of the sound. Over the timeframes, the temporal variations in sounds are represented. Many aspects of the ENI can affect the quality of these two essential tasks. To code the frequency spectrally (it could also be done temporally but with a more limited range), different SGCs need to be stimulated depending on the frequency of the sound. However, if the placement of the electrode array is subpar and/or the impedance is elevated by the fibrotic tissues, it is difficult to achieve precise stimulations. To code the intensity, sufficient SGCs need to be stimulated simultaneously. If the SGCs at the designated region are degenerated and/or lost, this task could be particularly difficult. Figure 1 demonstrates commonly discussed topics in ENI. To further understand the interactions between these factors of ENI, researchers proposed several important attributes alongside methods to measure them.

#### Channel independence

To be able to separate out different spectral regions, different populations of SGCs should be stimulated when different CI channels are activated. For CI users, speech perception improves with the number of independent channels up to seven or eight channels for the older generations^15,16^ and sometimes even more channels up to twenty for the newer models and recipients with better residual hearing prior to implantation^17–19^. Unfortunately, this can be challenging for CI hearing since the electrode array is surrounded by the highly conductive perilymph in the cochlea. The spread of excitation reduces channel independence between adjacent electrodes; cross-talk between spatially separate electrodes makes stimulation patterns difficult to distinguish despite different input signals^20,21^. Bierer^11^ described two main causes for increased spread of excitation, obstructed current path and electrode position (see Fig. 1). Cochlear implantation causes inflammatory responses in the soft and hard tissues in cochlea. Immune cells release cytokines and growth factors, which spread easily in the fluid-filled chamber, that signal fibroblasts to activate^22^. An analysis of 20 implanted human cochleae using 3D reconstructions reported that about 25% of cochlea’s volume was occupied by fibrous tissue and another 7% was occupied by newly formed bony tissue^23^. The fibrotic tissue encapsulates the electrode array increasing the resistance between the active and ground electrodes, resulting in the need for a higher potential difference (or current level, following the CI fitting software convention) to achieve sufficient auditory nerve stimulation^24^. The impedance typically increases shortly after implantation^24,25^ and stabilise over the course of electrical stimulation^26–29^. In some cases, the electrode array pierces the basilar membrane causing some contacts to sit in the scala vestibuli, which results in both fibrosis and sub-optimal electrode position and hence further elevates the resistance^30^. Similarly, if the distance between the electrode and the target SGCs is increased due to the electrode placement, a higher current level might be needed^31^. Altogether, the increased spread of excitation reduces the much-needed channel independence in CI hearing.

#### Neural health

The health of SGCs could be considered in two essential perspectives, the ability to code intensity and frequency. In typical hearing, the dynamic range for encoding loudness is achieved by different SGC populations, with the similar characteristic frequency (CF), having different firing characteristics and firing at different parts of the intensity range^32^. The frequency information is conveyed by both the CF of the firing population and the spiking rate of it^33^. However, this is challenging for CI hearing since the degeneration and or loss of SGCs, combined with the broad stimulation, makes precise control over the stimulation pattern and rate unachievable. Thus, the stimulated population is often comprised of many populations that convey different information in a typical hearing setting. The mechanism for loss and degeneration of SGCs is not fully understood but it is associated with ageing, deafness, hearing trauma, and exposure to ototoxic drugs^34–36^. The general trend of degeneration occurs from dendrites to the cell body^37,38^ and from the base to the apex of the cochlea^39^. The loss/degeneration of SGCs leads to fewer steps between the lowest and loudest perceivable loudness creating a narrower dynamic range that could be challenging in speech perception^40^. In some cases, the total loss of SGCs within a frequency band creates a ‘dead region’ (a spectral hole) on the frequency map^41,42^. Increasing current level would not resolve the dead region but introduce off-frequency hearing. That is, the stimulated SGCs are not the intended recipients determined by the CI coding strategy. In addition, the elevated current level needed due to the loss/degeneration of SGCs can affect the channel independence.

#### Insertion depth and frequency to place mismatch

The SGCs are organised tonotopically (Fig. 1). The ones with a lower CF are located at the apical part of the cochlea^43,44^. The electrode array is typically inserted into the Scala Tympani with an insertion depth (commonly measured in mm away from round window or degree angle starting from 0 clockwise) around 360–540 degrees covering only a portion of the whole 2.5 turns of the healthy cochlea^45^. Thus, the depth of insertion could affect the distance between the electrode and the designated SGC population. The difference between the CF of a channel and the CF of the stimulated SGCs is often referred to as the Frequency-to-Place Mismatch (FTPM). FTPM is often estimated using imaging tools (as discussed below) or electrophysiological methods^46^.

#### Viability

In this review, we refer to the term, viability of the ENI, as of the effectiveness of information transmission due to neural, non-neural and mixed factors (e.g., the health of the auditory nerve, electrode placement and the spread of excitation due to both). The more “viable” the ENI is, the better the information transmission will be.

### Estimating the viability of ENI

A range of approaches have been proposed to estimate the viability of the ENI. Such approaches involve imaging techniques, psychophysical measures (subjective, requiring patients to be able to do the task and respond), and electrophysiological (objective) measures. It is impractical to directly measure the status of the ENI without causing damage. Thus, there are always compromises and trade-offs involved in the proposed ENI measures. In the following paragraphs, approaches are introduced first and followed by the applications on CI mappings.

#### Imaging tools

Imaging techniques including X-ray and Computed Tomography (CT) with various resolutions are used to estimate the electrode-to-modiolus distance (EMD), determine the location of the electrode, and diagnose insertion trauma^12,31,47–56^. Knowing these attributes, researchers proposed methods of estimating the FTPM and channel interactions.

#### Psychophysical measures of ENI viability

The psychophysical measures of the ENI use detection and discrimination tasks that can explain and quantify degrees of channel independence and the effectiveness of processing sounds falling within the same channel.

Several electrode discrimination tasks have been used to locate electrodes or regions of electrodes that are not perceptually distinct from one another^57–61^. The common paradigms often include stimulating different electrodes to test if they are discriminable^57,61^. Another paradigm involves a pitch ranking task sequentially stimulating a pair of electrodes and ask the participant to indicate the one with a higher pitch. An pair of electrodes is considered discriminable if the correct rate of identification is reached within certain number of trials^58–60^. In this review, we refer both paradigms as to an electrode discrimination task despite the difference between identification (stating high as high is considered correct) and discrimination (stating the difference itself warranties correctness). An indiscriminable electrode potentially suggests significant stimulation overlap with neighbouring electrodes. It is worth noting that discrimination tasks could also be cued by the change of loudness. As a result, the successful discrimination between electrodes could be partially informed by the change of loudness instead of the change of perceived pitch.

(Frequency) tuning curves have been used to estimate channel interactions in several studies^62–64^. The probe-detection thresholds for different masker-probe combinations are plotted to form the tuning curve indicating the change in magnitude of the threshold for each masker. This provides a measure of frequency selectivity and broadness of current spread.

Zhou^65^ reported that the detection threshold at a lower pulse rate, 80 pulses per second (pps), correlated more closely (adjusted R squared of 0.526) with the spatial selectivity (measured with a forward masking paradigm such as the tuning curve method) compared with the threshold measured at the conventional stimulation rate of 900 or higher pps and hence proposed the use of low rate threshold as a practical option of measuring the spatial selectivity.

Tripolar stimulation was proposed to enhance the spatial selectivity by applying a biphasic pulse to one stimulation electrode, and applying an opposite polarity biphasic pulse (reduced in magnitude) to the two electrodes to the right and left of the stimulation electrode. Bierer^66^ measured detection thresholds using tripolar stimulation and found a correlation between the performance on a clinical speech test and the variability in tripolar threshold across electrodes. It was suggested that higher tripolar thresholds were indicative of a poorer neural survival or electrode placement.

Changing the pulse-rate of an electric pulse train at one electrode changes the voltage needed to elicit an audible sensation. Multipulse-integration (MPI) is a measure proposed to describe neural health at a particular electrode site (within channel). It is measured by estimating the threshold differences between pulse trains with different rates at a fixed duration. Normally, it is reported in terms of slope (change in dB/ change in rate) and a steeper slope reflects better neural survival when studied with animal models^67–69^. In these animal models, it is hypothesised that a site with better neural health (higher population of SGCs and in better status) should be able to respond to the additional pulses better and hence demonstrate a lower threshold. Zhou and Dong^70^ measured MPI with CI recipients and found that MPI correlated with the spread of excitation measured as the slope of the tuning curve.

The Polarity Effect, the difference between detection thresholds due to the polarity of biphasic or multi-phasic pulses (e.g., anodic versus cathodic first), has been proposed to reflect the status of the neural health. Based on modelling works, it is predicted that the anodic leading pulses excite more degenerated or demyelinated peripheral processes of the human auditory nerve^71,72^. Thus, polarity effects could be a marker of neural degeneration. Studies with human CI users reported this finding consistently over recent decades^73–75^. Furthermore, this effect can also be observed objectively through electrophysiological methods^76^.

#### Electrophysiological measures of ENI

Electrically-evoked compound potentials (eCAP), are the temporal responses from a group of SGCs recorded directly from the CI electrodes^77^. A typical eCAP recording shows a valley followed by one peak (a N1-P1 response). With the increasing stimulation level, the amplitude grows correspondingly. To determine if a response is present, the morphology of the eCAP is analysed. However, the correlation between the eCAP threshold and behavioural thresholds is moderate with a Pearson’s r around 0.5–0.6^78–80^. The challenge is potentially related to the eCAPs being measured at a much lower stimulation rate (e.g. 30 Hz) than the clinical coding strategy rate (around 900 pps or higher). eCAP could be used as an alternative to the behaviour response. For instance, polarity effects have been explored with eCAP recordings^76^. In addition to being an objective alternative, eCAP are used in algorithms that estimate the ENI status automatically. For instance, tuning curves constructed by eCAP recordings across the array with an algorithm modelling the effect of neural health and current spread has been demonstrated^81,82^. In addition, analysis of eCAP recordings was proposed to identify spread of excitation and was reported to correlate with speech perception^83^.

Several eCAP studies reported that the amplitude grows as a function of the gap between the two phases of a pulse (termed interphase gap effect, IPG). Studies with rodents^84,85^ have shown a correlation between the IPG effect and neural density. Similarly, studies with CI users showed correlation between speech perception and the IPG effect.

### Interventions informed by ENI

If the ENI is extensively damaged it could be a bottleneck for the transmission of sound^14^. Many of the measures that have been explored have not yet been used to re-map CIs but all ENI measures indicate high variability between participants and, more importantly, within participants across electrodes. However, clinicians often use the same approach to mapping for all patients: eCAP-Thresholds for infants, and uncooperative adults, subjective comfortable and threshold values for the reminder of patients while electrodes causing pain or facial nerve stimulation are reduced in stimulation level or disabled. This approach is efficient clinically, yet, leaving room for improvement. The mapping approaches informed by an ENI measure that have been investigated are typically either electrode deactivation or frequency re-allocation of the channels.

#### Electrode deactivation

Several publications showed the result of deactivating electrodes with a poor ENI^56,58–60,86–96^. For a typical electrode deactivation intervention (also termed site selection strategy), the researchers estimate the ENI status of each electrode and deactivate the ones with poor ENI status. The tool adopted to estimate ENI and the rules used to deactivate electrodes varies between approaches. Yet, they shared the same objective of reducing channel interactions assuming channels with poor ENI status are more likely to introduce distortion, off-frequency listening and interference.

#### Adjustments of frequency allocation

Changes of the frequency allocation table were seen in several articles. Two articles tailored the frequency allocation for each CI user based on responses from discrimination tasks^97,98^. Others aimed at reducing FTPM to have a better match to the CF of the stimulated SGCs^48–50,52,55,99–101^. Interventions of this type starts with an estimate of electrode location. Next, the location is transformed into CF frequency using different mapping functions^43,44^. Lastly, the frequency allocation is changed aiming to reduce the FTPM.

### Research objectives

It is unclear which intervention might be most helpful for the different ENI issues. To identify promising interventions, this review evaluated research articles on re-mapping that were informed by ENI measures. A Participant, Interventions, Comparison, Outcome, Study (PICOS^102^) method was adopted to answer the following question:

Do interventions informed by individual ENI measures improve speech outcomes in adult CI users compared with the clinical settings?

A meta-analysis was conducted to quantify the key findings of the included articles. In addition to the main question, the interaction between ENI measures and interventions was also explored.

## Method

### Data sources and inclusion criteria

**Fig. 2:**
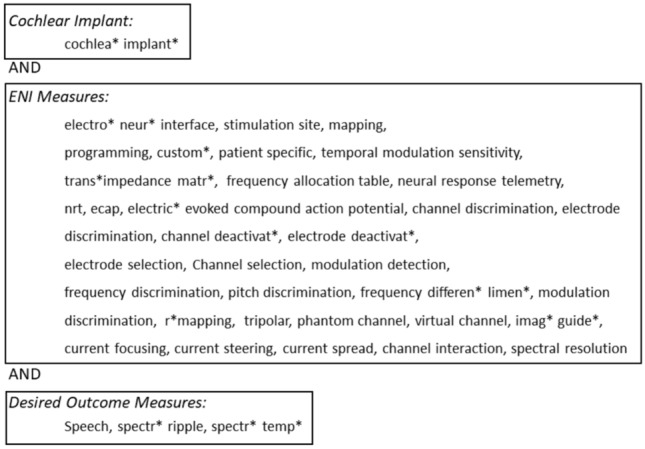
Search terms. The asterisk stands for any character in the search bar (e.g., implant* gives results from implant, implantation … etc.). The terms are separated by a comma. Within each box, terms are combined with the Boolean OR. Between boxes, terms are combined with the Boolean AND.

A systematic review registered with PROSPERO (international prospective register of systematic reviews, ID CRD42021292483) was performed in accordance with the PRISMA 2020 statement^103^. The study protocol was designed and registered before starting to ensure the transparency. Six databases, MEDLINE, EMBASE, TRIP, Scopus, Web of Science, and CINAHL, were searched in April 2024. Inclusion criteria for articles were guided by the PICOS question. Participant refers to the population of interest. Adult CI users were selected because these are the group who can reliably respond in psychophysical tasks; also, most literature on remapping has been conducted with adults. Interventions looked at changes in CI settings and training regimes that were specifically informed by ENI measures, not requiring surgical or pharmaceutical approaches. Comparator was set as the daily-used clinical map to provide a scenario closer to the practice. Outcomes of interest were speech perception in quiet and noise and other correlated tests (e.g., spectral ripple, spectral-temporally modulated test). Studies to be included were restricted to primary human studies so that each experiment contributes as one piece of evidence only. The search term for the databases was defined by the combination of CI, ENI measures, and desired outcome measures (Fig. 2). See appendix A for the exact search terms used in different electronic databases.

### Selection process

Duplicates were removed from the articles arising from the search. The list was processed against the inclusion criteria by the corresponding author using titles and abstracts. The eligible publications then went through the data extraction and Risk of Bias (RoB) assessment. Each reviewer had the opportunity to exclude a publication during the RoB assessment if a previously missed detail justified the removal of a study (four were removed at this stage).

### Risk of bias assessment

Three reviewers (JL, BW, DV) conducted the assessment independently. A questionnaire was tailored to consider the strength of evidence from the articles (see appendix B for the questionnaire and marking guideline). Questions tailored to CI intervention studies were created and some questions addressing the research question, confounds, and participant recruitment based on the CASP (Critical Appraisal Skills Programme) checklists were adopted^104^: Each article was first assessed using nine domains (Study Design, Participant Selection, Presence of Control Group, Learning Effect, Adaptation/Acclimatisation Period, Outcome Measure, Statistical Biases, Conflict of Interest, and Reporting) and a global score was assigned based on the responses to each aspect. The marking rationale was adapted from ROBINS-I (for comparing the intervention study with a target trial (ROBINS-I by Cochrane^105^). The target trial was an imaginary trial that answers the same research question in the best way possible. If the assessed article answers all questions in a domain comparably to the target trial, it is marked as a low RoB for that domain. Possible scores were low, moderate, and high. Whenever the three reviewers’ responses contained one each of the extremes (high and low), this counted as a conflict and the findings were discussed to reach consensus. For all other cases the most frequently occurring score was used. The global score was determined by the following rules, applied one after the other. Rule 1, if 2 or more domains were marked ‘High’, the global score was marked ‘High’. Rule 2, if the first rule didn’t apply, the global score was marked ‘Low’ when six or more domains were marked ‘Low’. Rule 3, if the two previous rules did not apply, the global score was marked ‘Moderate’.

### Data extraction

The corresponding author extracted the following data items: study design, sample size (in number of ears and participants), intervention approach, adaptation period (to a new map), details of the comparator, speech in noise performance, and speech in quiet performance. For speech in noise outcomes, articles commonly reported dB SNR (signal-to-noise-ratio) needed for 50% correct, or the percentage correct at one or more SNRs tested with sentences and/or words. If results were reported for both sentence and word tests, the score of the sentence test was used for analysis. In the case where unilateral and bilateral results were both reported, the unilateral result was extracted. The mean and standard deviation of improvements were extracted. If the article did not report them explicitly, the corresponding author inferred the standard deviation (SD) using the reported statistics when applicable (E.g., using the number of participants, mean difference, and T-statistics to infer the SD). To obtain individual data, a web-based application (WebPlotDigitizer v4) was used to extract data points from the published figures^106^. The corresponding authors were contacted for raw data if the key statistics could not be computed with the extracted data alone.

### Study designs

CI mapping studies are a flexible intervention approach in medical research. Unlike many pharmaceutical or surgical interventions, the change of map is reversible in most cases. The clinical map was typically the control condition, and the experimental map was the intervention. The designs that compared the performance of maps within the same participant were classified as ‘Crossover’ if more than one experimental intervention was tested or ‘Single Intervention’ if only one condition was trialled per participant. ‘RCT’ was added if the study design included a control group, and the group assignment was randomized. ‘Longitudinal Single Intervention’ study measures the outcomes at different times from the same group of participants. ‘Cross-sectional’ study is a type of observational study that measures the outcomes at one point in time on the group of participants meeting the inclusion criteria.

### Data synthesis

Included articles differed largely in outcome measures, study design, intervention method and publication year. Therefore, a random-effects model was adopted for the meta-analysis to compute a standardised mean difference (Cohen’s d) and weighting the outcomes using both the within and between study variances^107–109^. In this view, we believe that the included articles were different (in ENI measures, interventions, and precision due to study design) but all fall under the definition of interventions informed by ENI measures. To conduct such synthesis, the mean difference (improvement, positive being better) and standard deviation of the differences (SD_d_) were needed. For self-controlled studies, the SD_d_ was directly computed from the raw data when possible or inferred from the t statistics. For RCT studies, the SD_d_ was computed as the pooled SD between the control and experimental groups.

Meta-regressions were conducted to further explore the interplays between the interventions and ENI measures. If a study was marked having a high risk of bias or the mean and SD were unavailable, the study was excluded from the meta-analysis. Computations were conducted using a custom script written in Python3 with SciPy version 1.10.1^110^ and NumPy version 1.26.4^111^. In addition, publication bias was analysed using a funnel plot.

## Results

**Fig. 3:**
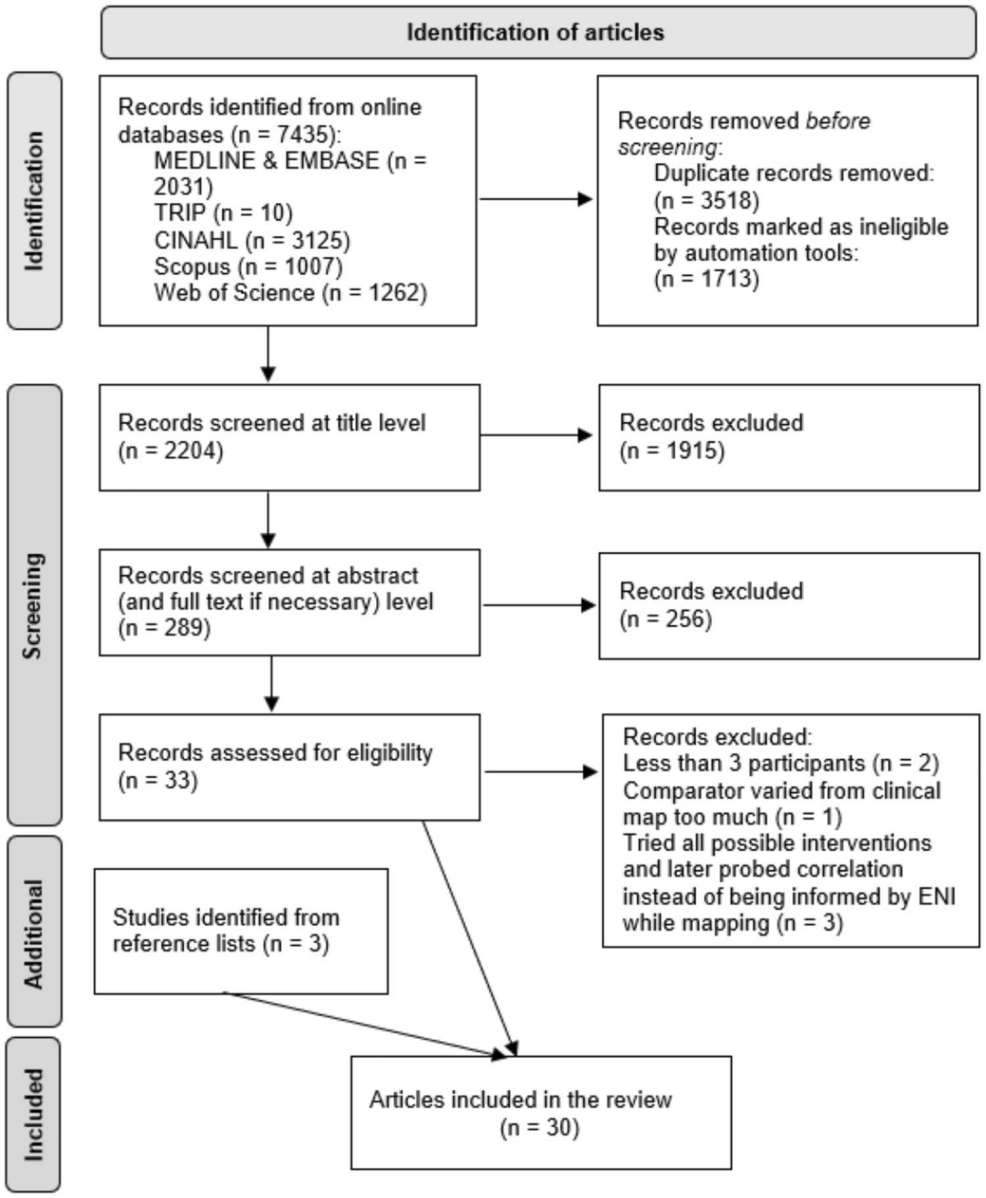
Consort diagram for article identification. Gray boxes mark each stage of identification. The flow of articles is indicated by the arrows. The first column of white boxes demonstrates the articles remained in each stage. The second column of white boxes show the removed articles.

The search resulted in 7435 articles being identified from the six electronic databases. Duplicated (n = 3518) and ineligible records (n = 1713) were removed leaving 2204 articles for screening at title level. After the title screening, 289 articles remained and were checked at the abstract or full-text level to assess relevance. Thirty-three articles remained of which six were further excluded due to having a small sample size^101,112^, adopting a comparator that is too different from the modern clinical map^113^, or attempting several interventions and later probe the relationship of the ENI measure instead of being informed while mapping CI^98,114,115^. Three additional articles were identified from reference lists resulting in 30 included articles (Fig. 3) in total^48–50,52,54–56,58–62,86–97,99,100,116–119^.

### Profile of the included articles

The characteristics and key findings of the included articles are summarised in Table 1. If a study experimented on more than one intervention map (excluding the inverse controls), the outcome of each map was reported in separate rows. The outcome measures were typically speech tests and these included word, consonant, vowels, and sentences (in noise and quiet). Sentence tests typically adopted, BKB, CUNY, HINT, and IEEE sentences. Speech in quiet measures often consisted of consonant–vowel-consonant and vowel-consonant–vowel tests. The number of participants varied from 3 to 72 (Mean = 14, SD = 13). Most participants were post-lingually deaf adult CI users while two articles^62,63,91^ included pre-lingually deaf adults (N = 2 and 26 ears respectively) and one article included EAS users^49^. All but two studies adopted a within-subject paradigm to compare the performance between pre-intervention and post-intervention. Single blinding was rare, and two studies were randomised control trials. Different adaptation periods were seen ranging from several months to acute testing within one session. Most articles fell into two re-mapping areas, (1) channel deactivation to reduce channel interaction (16 articles), and (2) adjustments of frequency allocation (12 articles). Other interventions included increasing T-level^119^, using a new coding strategy^117^, change of stimulation mode^62^, and dichotic but complementary site selection^118^.

**Table 1:** Key findings of the included articles.

Study	ENI measure	Intervention	Study design	Adaptation period	RoB	SiN improvement (Effect Size)	SiN improvement (Original Scale)	Standard deviation (SiN Improvement)	SiN sample size	SiQ improvement (Effect Size)	SiQ improvement (Original Scale)	Standard deviation (SiQ Improvement)	SiQ sample size
Goehring et al.^89^	Polarity Effect	Deactivation	Crossover	Acute	Low	− 0.42	− 1.24	2.99	8	− 0.69	− 5.18	7.53	8
Jiam et al.^100^	CT Image	reduce FTPM	Single Intervention	Acute	Moderate	0.26	0.69	2.63	16	0.22	0.02	0.09	16
Danieli et al.^87^	CT Image	Deactivation	Single Intervention	4 weeks	High	0.40	13.00	32.19	10	0.36	12.00	33.50	14
Noble et al.^92^	CT Image	Deactivation	Single Intervention	3–6 weeks	Moderate	0.02	0.03	2.01	72	0.08	1.18	15.50	72
Saleh et al.^58^	Electrode Discrimination	Deactivation	Crossover	4 weeks	Moderate	0.63			25	0.51			20
DeVries and Arenberg^62^	CT_Image	Focused Stimulation	Crossover	Acute	Moderate	0.45	4.95	10.99	9	0.29	3.26	11.18	4
DeVries and Arenberg^63^	Tuning Curve	Focused Stimulation	Crossover	Acute	Moderate	0.36	2.85	7.98	9	− 0.08	− 0.84	10.11	4
Vickers et al.^59^	Electrode Discrimination	Deactivation	Crossover	Long term	Low	No Difference			13	No Difference			13
Zhou^95^	Low-Rate Threshold	Deactivation	Single Intervention	Acute	Moderate	1.83	4.03	2.20	8				
Zhou and Pfingst^69,119^	Modulation Detection Threshold	Increase T Level by 5% Dynamic Range	Crossover	Acute	Moderate	1.34	2.38	1.78	9				
Noble et al.^93^	CT Image	Deactivation	Single Intervention	3–4 weeks	Moderate	0.83	3.00	3.63	10	0.69	12.73	18.37	11
Falcón González et al.^97^	Behavioural Instrument Discrimination Task	Frequency re-allocation	Observational	4 weeks	High	roughly 10 dB SNR improvement for HINT reported by the author			20				
Zhou^96^	Low-Rate Threshold	Deactivation	Longitudinal	8 weeks	Low	1.52	4.66	3.07	9	1.04	10.94	10.53	9
Grasmeder et al.^99^	X-Ray	reduce FTPM, SG Map	Crossover	6 weeks	Low	− 0.98	− 11.65	11.94	10				
Grasmeder et al.^99^	X-Ray	reduce FTPM, Greenwood Map	Crossover	6 weeks	Low	− 3.70	− 68.70	18.55	10				
Zhou and Pfingst^118^	Modulation Detection Threshold	Complementary Bilateral Site Selection Map B	Crossover	Acute	Moderate	1.41	1.77	1.25	8				
Bierer and Litvak^86^	Partial Tripolar Threshold	Deactivation	Crossover	Acute	Moderate	0.33	2.32	7.04	9	− 0.05	− 0.54	10.46	9
Labadie et al.^91^	CT_Image	Deactivation	Single Intervention	3–6 weeks	High	− 0.29	− 1.22	4.15	25	− 0.02	− 0.33	14.05	
Garadat et al.^88^	Modulation Detection Threshold	Deactivation	Single Intervention	Acute	High	0.09	1.58	17.45	12	0.30	5.44	18.44	12
Schvartz-Leyzac et al.^94^	ECAP IPG Effect	Deactivation	Crossover	Acute	Low	0.07	0.10	1.54	18				
Henshall and McKay^90^	Multidimensional Pitch Scaling	Deactivation	Crossover	2 weeks	Low	− 2.03	− 2.13	1.05	3	1.29	2.42	1.88	2
Tabibi et al.^117^	ECAP Refractory Period	Coding Strategy Informed by individual ANF’s characteristics	Single Intervention	Acute	Moderate	− 1.07	− 3.05	2.85	11				
Zwolan et al.^61^	Electrode Discrimination	Deactivation	Single Intervention	Acute	High	Seven of the nine participants showed significant improvement on at least one of the four speech recognition tasks tested							
Dillon et al.^50^	CT Image	reduce FTPM, SG Map	Crossover	Acute	Moderate					− 0.09	− 0.74	8.61	9
Dillon et al.^50^	CT Image	reduce FTPM, OC Map	Crossover	Acute	Moderate					0.18	1.76	9.67	9
Di Maro et al.^48^	CT Image	reduce FTPM	Observational	6 weeks	Moderate	0.94	10.00	10.61	10				
Fan et al.^52^	CT Image	reduce FTPM	Randomised Control Trial	52 weeks	Low	2.10	6.05	2.88	Con. 23, Exp. 25	1.51	3.52	2.33	Con. 23, Exp. 25
Dillon et al.^49^	CT Image	reduce FTPM with EAS users	Randomised Control Trial	24 weeks	Moderate	Inteventino Group N = 5 at the moment, showing some promising results see Table 3 of this article							
Dirks et al.^116^	Binaural Temporal Envelope Sensitivity with SSD CI users	reduce FTPM	Single Intervention	24 weeks	Low	− 0.60	− 0.59	0.98	7				
Warren and Atcherson^60^	Electrode Discrimination	Deactivation	Single Intervention	3–6 weeks	Moderate	1.95	13.38	6.86	12	0.67	4.03	5.99	13
Lambriks et al.^55^	CT Image	reduce FTPM	Single Intervention	6 weeks	Low	Median dB SNR 50% was significantly worse by 1.65 dB (Wilcoxon signed rank test, *p* = 0.03)			13	Median % correct dropped significantly by 10.1% (Wilcoxon signed rank test, *p* < 0.01)			14
Kurz et al.^54^	CT Image	reduce FTPM	Single Intervention	12 weeks	Moderate	0.95	0.75	0.79	10	0.55	3.67	6.72	13
McRackan et al.^56^	CT Image	Deactivation	Single Intervention	4 weeks	Moderate	− 0.19	− 0.49	2.61	17	0.17	1.41	8.21	17

This table summarises the key findings of the 30 included studies. SiN = speech in noise. SiQ = speech in quiet. Unavailable information was left blank. A positive value at SiN (Effect Size) or SiQ (Effect Size) represents a better performance (E.g., + 2 means 2 standard deviation smaller SNR SRT or higher percentage correct). RoB = Risk of Bias. CT = Computed Tomography. FTPM = Frequency to Place Mismatch. SG/SGC = Spiral Ganglion Cell. T-Level = Threshold Level.

### Risk of bias results

**Fig. 4:**
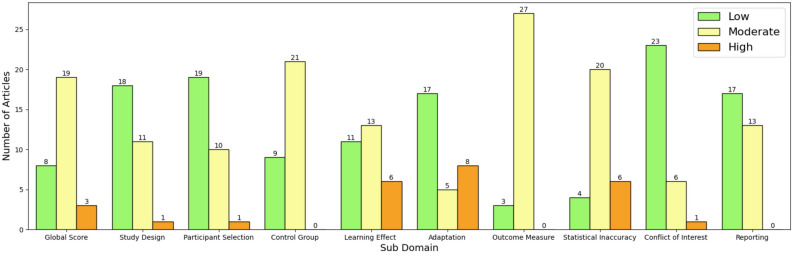
Risk of bias scores by sub-domain. For each sub-domain, the left (green), middle (yellow), and right (orange) bars represent the number of articles marked low, moderate, or high risk of bias, respectively.

Table 2 presents the RoB outcomes, including the scores of each sub domains for each article. At global level, among the 30 included articles, 19 were marked moderate, 8 were marked low (good quality), and 3 were marked high. Adaptation to the new map was the sub-domain that was most frequently (10 out of 30) marked having a high risk of bias. This result highlighted the need to consider a randomised control trial or to ensure the compared map has similar exposure as the clinical map. Statistical inaccuracy and learning effect were also marked high more frequently (6 out of 30). Some articles were considered high risk due to not reporting the statistical result in-full. In addition, learning effect could introduce biases if the testing order of maps was not randomised. Figure 4 demonstrates the distributions of scores by sub-domain. Overall, most included articles were considered to have a moderate or low risk of bias, which justifies the inclusion to the meta-analysis.

**Table 2:** Risk of bias results by domain.

Risk of Bias outcomes									
Study	Study design	Participant selection	Control group	Learning effect	Adaptation	Outcome measure	Statistical inaccuracy	Conflict of interest	Reporting
Goehring et al.^89^	Low	Low	Low	Low	Moderate	Moderate	Moderate	Low	Low
Jiam et al^100^.	Low	Low	Moderate	Low	Moderate	Moderate	Moderate	Low	Low
Danieli et al.^87^	High	Moderate	Moderate	High	Low	Moderate	High	High	Moderate
Noble et al.^92^	Low	Low	Moderate	High	Low	Moderate	Moderate	Low	Moderate
Saleh et al.^58^	Low	Low	Moderate	Moderate	Low	Moderate	Moderate	Low	Moderate
DeVries and Arenberg^62,63^	Low	Low	Low	Moderate	High	Moderate	Moderate	Low	Low
Vickers et al.^59^	Low	Low	Low	Moderate	Low	Moderate	Moderate	Moderate	Low
Zhou^95^	Moderate	Moderate	Low	Moderate	High	Moderate	Moderate	Low	Moderate
Bournique et al.^115^	Low	Low	Moderate	Low	Moderate	Moderate	Moderate	Low	Low
Zhou and Pfingst^69,119^	Low	Moderate	Moderate	Low	Moderate	Moderate	Moderate	Low	Moderate
Başkent and Shannon^114^	Moderate	Moderate	Moderate	Moderate	High	Moderate	Moderate	Low	Moderate
Noble et al.^93^	Low	Low	Moderate	Moderate	Low	Moderate	High	Low	Low
González et al.^97^	Moderate	Moderate	Moderate	High	Low	Moderate	High	Low	Moderate
Zhou^96^	Low	Low	Moderate	Low	Low	Moderate	Moderate	Low	Low
Grasmeder et al.^98^	Low	Low	Moderate	Moderate	High	Moderate	Moderate	Low	Low
Grasmeder et al.^99^	Low	Low	Low	Moderate	Low	Moderate	Low	Low	Low
Zhou and Pfingst^118^	Moderate	Low	Moderate	Moderate	High	Moderate	Moderate	Low	Moderate
Bierer and Litvak^86^	Moderate	High	Low	Low	Moderate	Moderate	Moderate	Moderate	Low
Labadie et al.^91^	Moderate	Low	Moderate	High	Low	Moderate	Moderate	Low	Moderate
Garadat et al.^88^	Moderate	Low	Moderate	Moderate	High	Moderate	Moderate	Low	Moderate
Schvartz-Leyzac et al.^94^	Moderate	Low	Low	Low	High	Low	Low	Low	Low
Henshall and McKay^90^	Low	Low	Moderate	Low	Low	Moderate	Moderate	Low	Low
Tabibi et al.^117^	Low	Low	Moderate	Moderate	High	Moderate	Moderate	Low	Low
Zwolan et al.^61^	Low	Low	Moderate	Moderate	High	Moderate	High	Low	Moderate
Dillon et al.^50^	Moderate	Moderate	Moderate	Moderate	High	Moderate	Moderate	Moderate	Low
Di Maro et al.^48^	Moderate	Moderate	Moderate	Moderate	Moderate	Moderate	Moderate	Low	Low
Fan et al.^52^	Low	Low	Low	Low	Low	Low	High	Low	Moderate
Dillon et al.^49^	Low	Moderate	Moderate	Low	Low	Moderate	High	Moderate	Moderate
Dirks et al.^116^	Low	Moderate	Moderate	Low	Low	Moderate	Low	Low	Low
Warren and Atcherson^60^	Low	Low	Moderate	Moderate	Low	Moderate	Moderate	Low	Low
Lambriks et al.^55^	Low	Low	Low	Low	Low	Low	Low	Moderate	Low
Kurz et al.^54^	Moderate	Moderate	Moderate	High	Low	Moderate	Moderate	Moderate	Moderate
McRackan et al.^56^	Moderate	Moderate	Moderate	High	Low	Moderate	Moderate	Low	Low

A low risk of bias indicates that the assessed article is considered having a comparable risk of a well-conducted randomised control trial.

### Publication bias

**Fig. 5:**
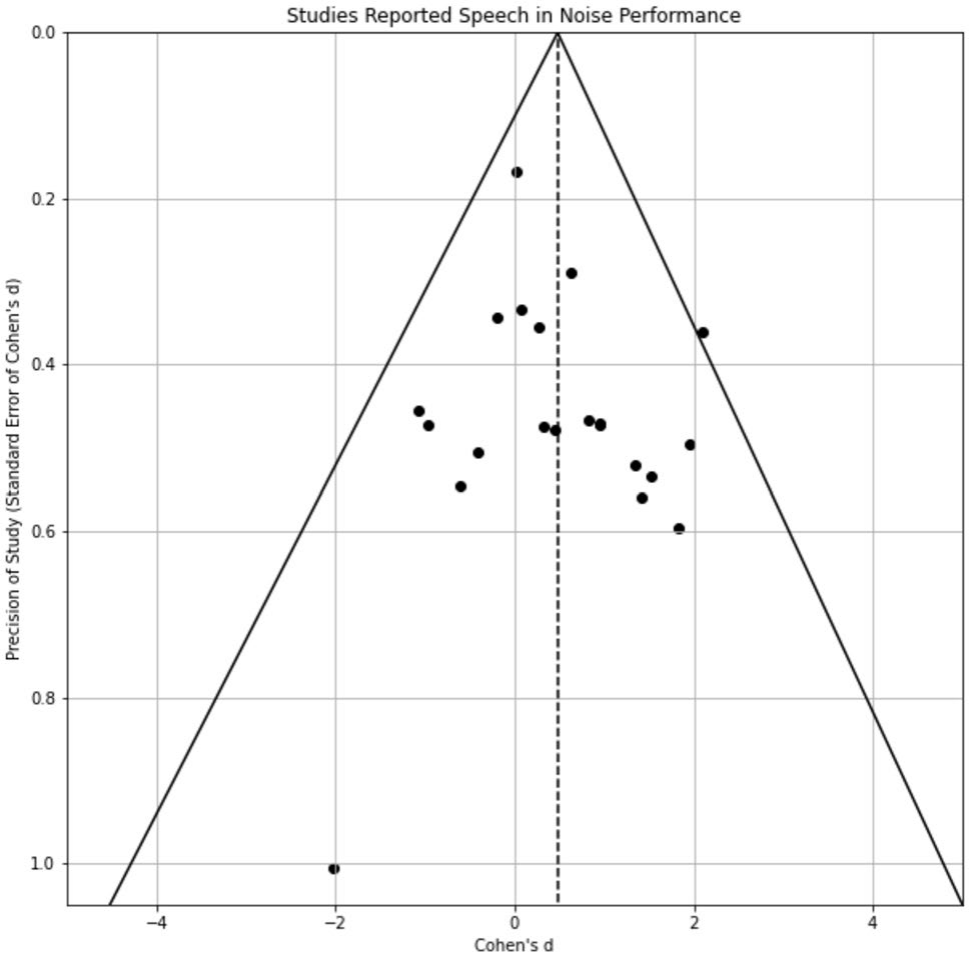
Relationship between the reported outcome and study precision. Y axis represents the precision of the study using the scale of standard error of Cohen’s d (smaller the standard error, higher the precision). X axis represents the reported effect size. Each black dot represents a study (more participants and smaller within-study variance give higher precision). The dashed line shows the combined Cohen’s d (0.56). This plot is often recognised as the funnel plot for assessing publication bias. If articles with a positive finding were favoured at publishing, there should be more studies on the right of the dashed line (combined Cohen’s d at 0.48). If a study is truly precise, the reported outcome should be closer to the ground truth, which should be around the combined effect size.

The funnel plot demonstrated symmetry between the studies reporting a Cohen’s d higher or lower than average (Fig. 5). An Egger’s test was conducted to test if there’s a relationship between the standardised effect size and the inverse of the standard error of the Cohen’s d. The result revealed a slope that was not significantly different from zero (r = 0.014, *p* = 0.95) and hence supporting it was more likely than not that the publication bias was not present.

### Meta-analysis

#### Do re-mapping interventions informed by ENI measures improve speech perception performance?

**Fig. 6:**
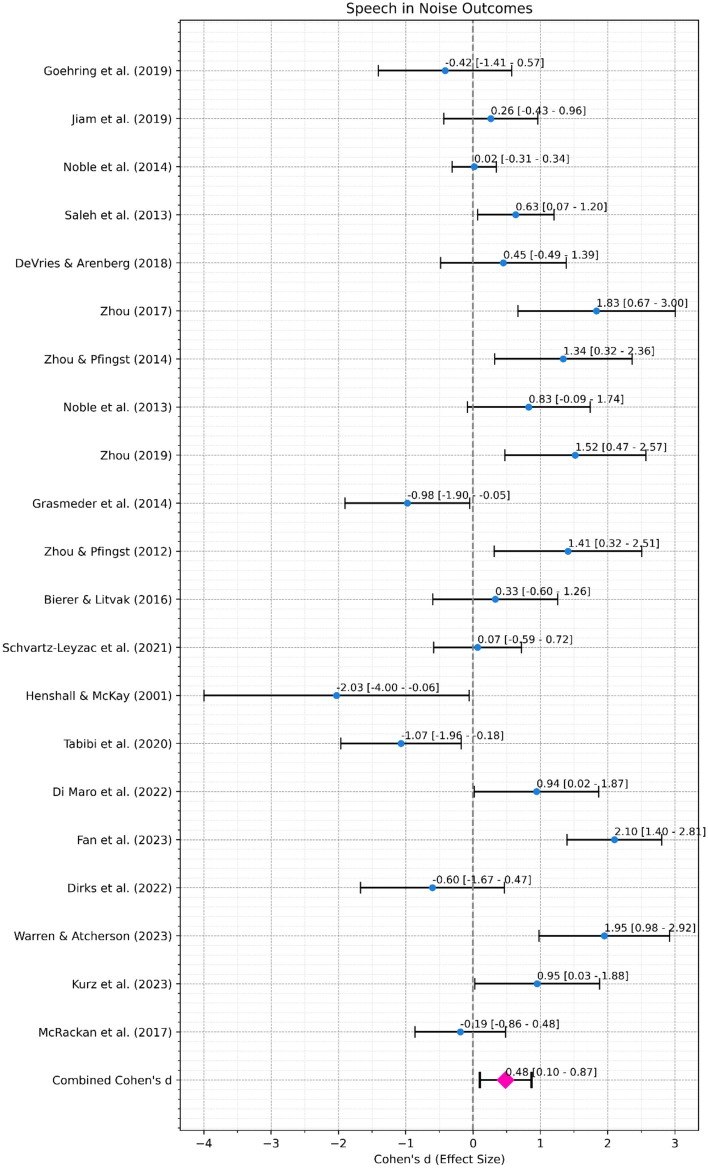

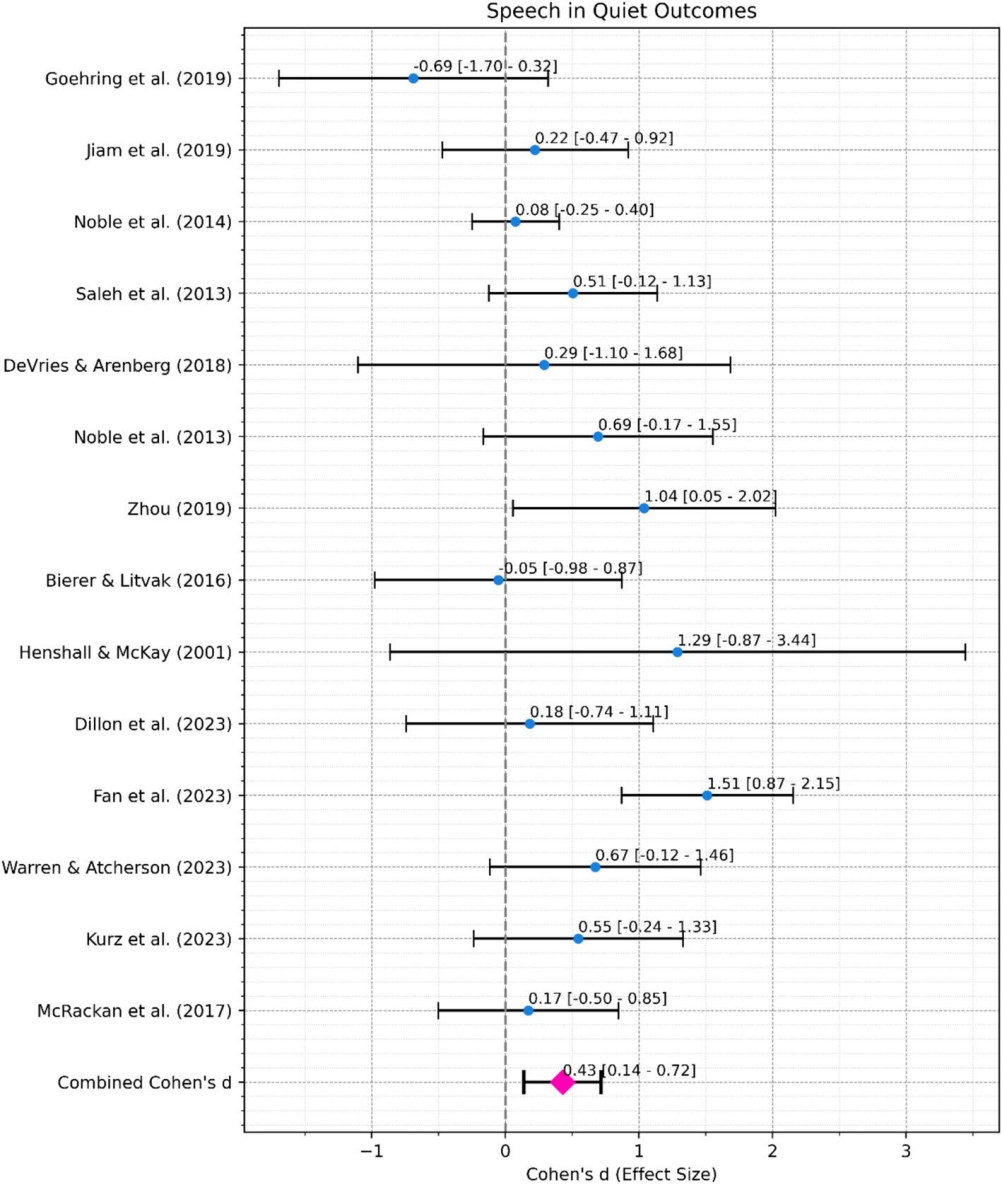
(**a**) Forest Plot for Speech in Noise Outcomes. Black error bars mark the 95% confidence interval. Blue dots mark the individual Cohen’s d. The pink diamond marks the combined Cohen’s d. (**b**) Forest Plot for Speech in Quiet Outcomes. Black error bars mark the 95% confidence interval. Blue dots mark the individual Cohen’s d. The pink diamond marks the combined Cohen’s d.

*Speech in Noise Performance*. A meta-analysis was conducted to assess the overall effect of re-mapping interventions informed by ENI measures on speech in noise performance (Fig. 6a). The combined effect size, from 21 studies with 305 ears in total, was 0.48 with a 95% confidence interval ranging from 0.1 to 0.87, indicating a moderate and statistically significant (*p* = 0.014) effect. Heterogeneity was significant (Cochran’s Q = 93.09, df = 20, *p* < 0.001) indicating differences in effect size across studies.

*Speech in Quiet Performance*. A meta-analysis was conducted to assess the overall effect of re-mapping interventions informed by ENI measures on speech in quiet performance (Fig. 6b). The combined effect size, from 14 studies with 227 ears in total, was 0.43 with a 95% confidence interval ranging from 0.14 to 0.72, indicating a moderate and significant (*p* < 0.01) effect. Heterogeneity was again significant (Cochran’s Q = 24.91, df = 13, *p* = 0.023) indicating differences in effect size across studies.

#### Does speech in noise performance improvement come at the cost of speech in quiet performance?

**Fig. 7:**
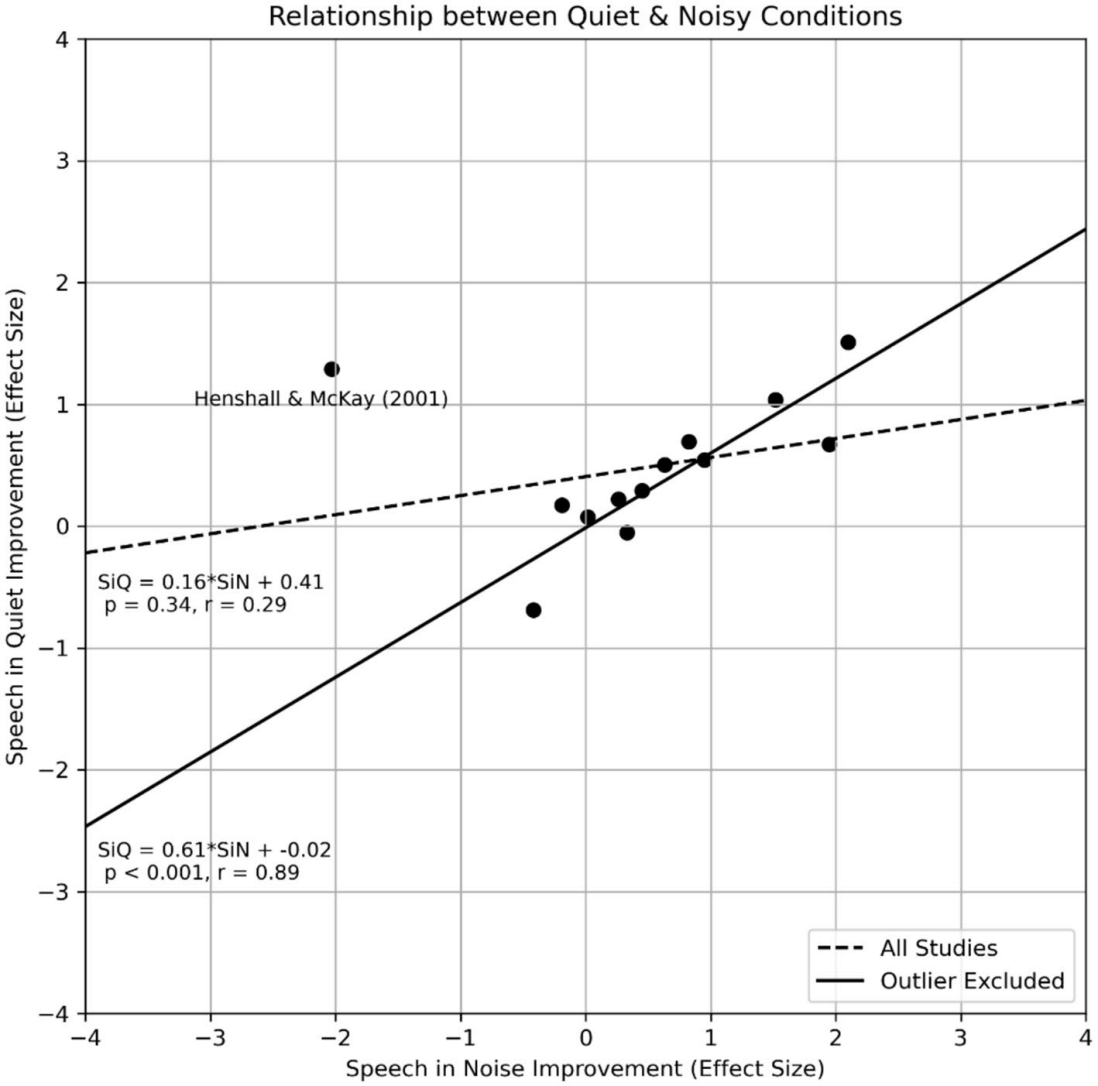
Relationship between speech in noise and speech in quiet performances. The axes represent the improvement provided by the intervention in effect size (positive is better); the dashed and solid lines are the line of best fit, with or without the outlier. Each black dot represents an article. The Henshall and Mckay^90^ paper was a leader in the field and three of the seven participants reported both speech in noise and quiet results while other studies were conducted ranging from 2013 to 2023 with a higher number of participants.

Clinicians would be interested in understanding if re-mapping results in an improvement in speech in noise perception; if so, are there any negative consequences, such as deterioration in speech in quiet performance. For instance, switching off electrodes with poor ENI viability could reduce channel interactions, but result in fewer active channels. A linear regression was conducted to assess the relationship between the speech in noise and quiet performances (Fig. 7). Among the 12 articles that reported performance for both speech testing conditions^52,54,56,58,60,62,86,89,90,92,93,96^, while one outlier was removed due to only three out of the seven participants were able to complete speech tests under both conditions, a positive and statistically significant correlation was seen between the average speech in noise and quiet performance (r = 0.89, *p* < 0.001) indicating that the performances tend to improve concurrently.

#### Performance of the site selection interventions

**Fig. 8:**
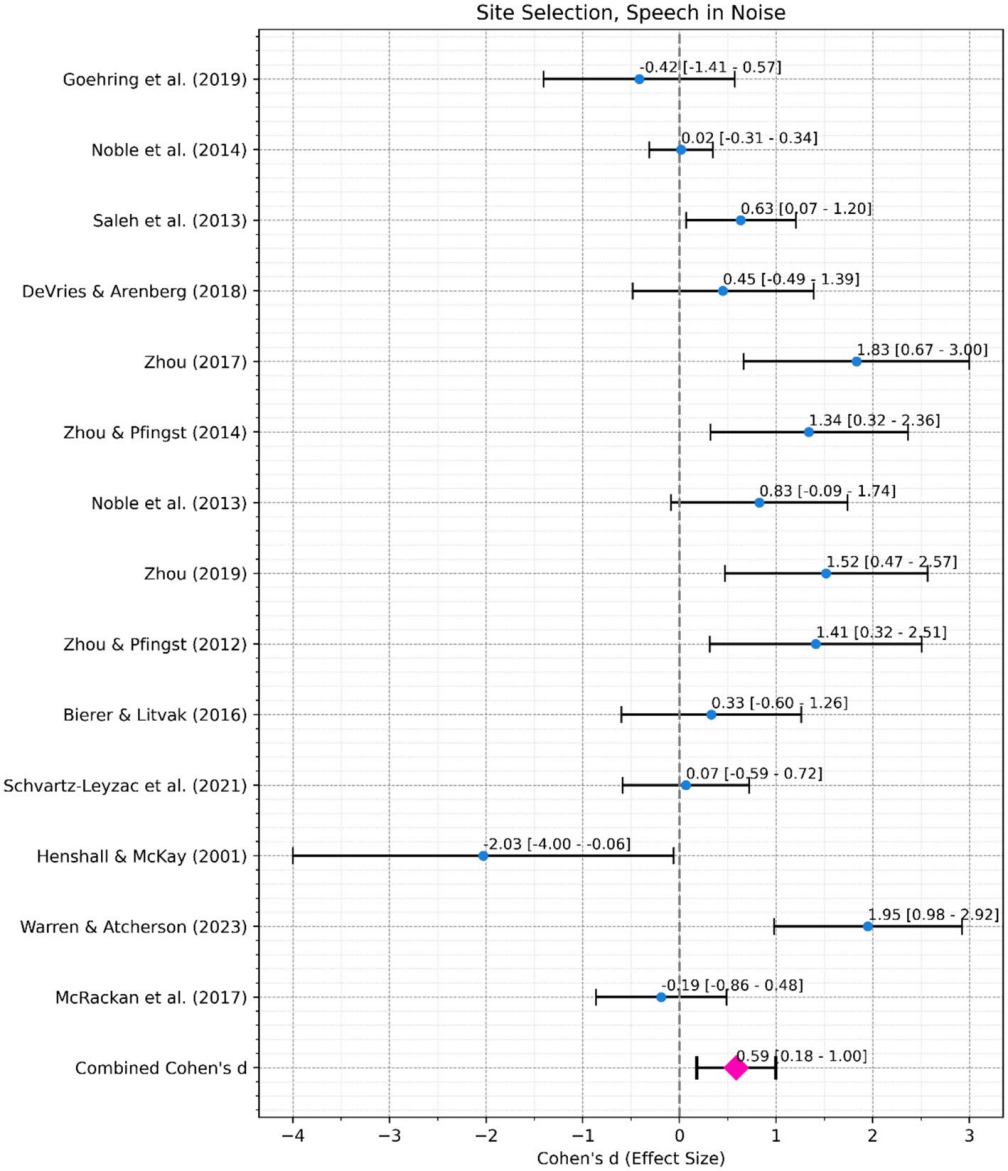

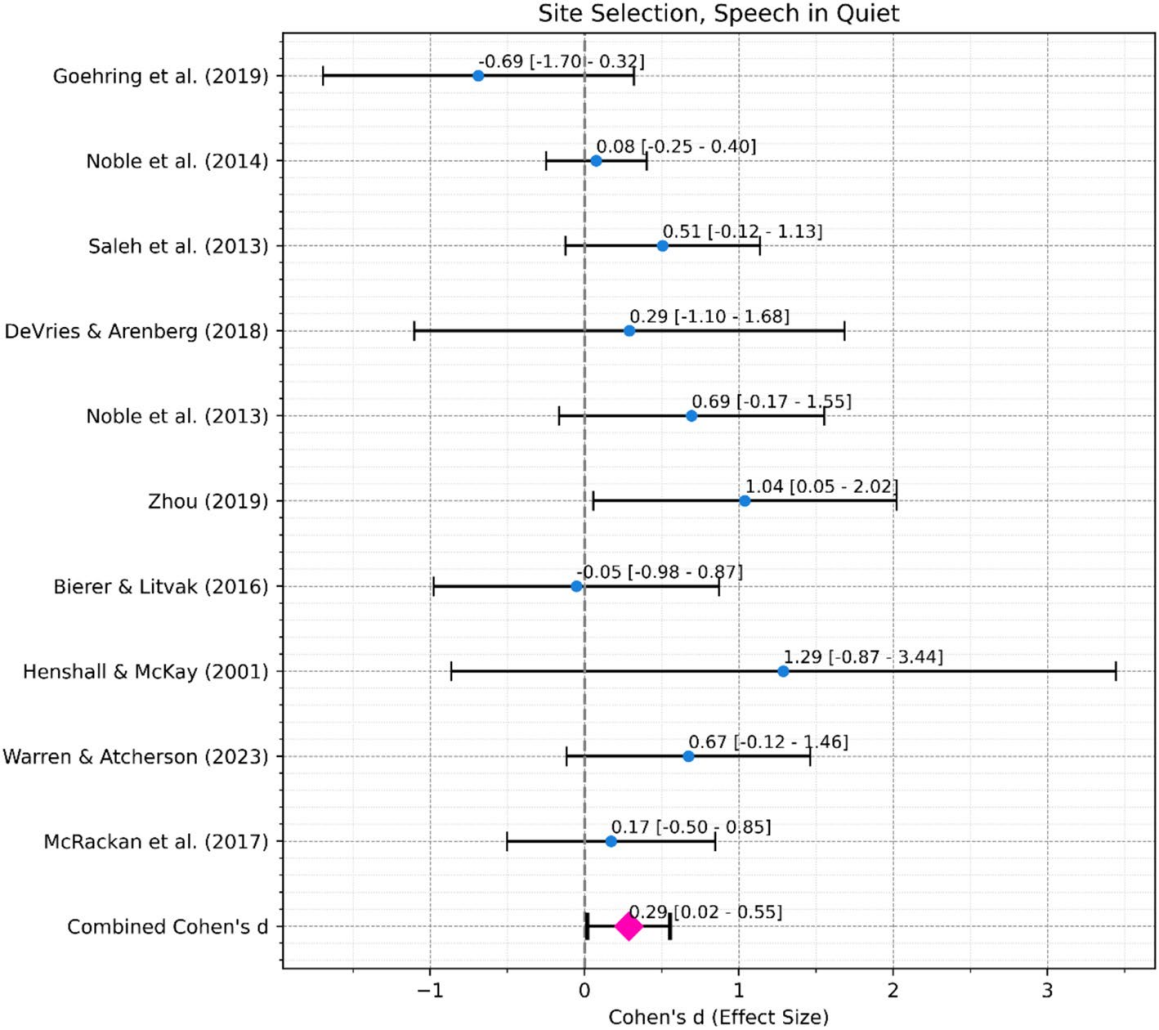
(**a**) Forest Plot for Site Selection Interventions, Speech in Noise. Black error bars mark the 95% confidence interval. Blue dots mark the individual Cohen’s d. The pink diamond marks the combined Cohen’s d. (**b**) Forest Plot for Site Selection Interventions, Speech in Quiet. Black error bars mark the 95% confidence interval. Blue dots mark the individual Cohen’s d. The pink diamond marks the combined Cohen’s d.

*Speech in Noise Performance*. A meta-analysis was conducted to assess the overall effect of site selection (deactivation) interventions informed by ENI measures on speech in noise performance (Fig. 8a). The combined effect size, from 14 studies with 217 ears in total, was 0.59 with a 95% confidence interval ranging from 0.18 to 1, indicating a moderate and statistically significant (*p* = 0.005) effect. Heterogeneity was significant (Cochran’s Q = 45.62, df = 13, *p* < 0.001) indicating differences in effect size across studies.

*Speech in Quiet Performance*. A meta-analysis was conducted to assess the overall effect of site selection (deactivation) interventions informed by ENI measures on speech in quiet performance (Fig. 8b). The combined effect size, from 10 studies with 165 ears in total, was 0.29 with a 95% confidence interval ranging from 0.02 to 0.55, indicating a small and significant (*p* = 0.036) effect. Heterogeneity was not significant (Cochran’s Q = 10.99, df = 9, *p* = 0.28).

*Differences between ENI Measures*. To explore whether site selection performs better while being informed by a specific ENI measure, a weighted least square regression was conducted using the effect size as the dependent variable, the ENI measure as the independent variable (categorical) and the inverse of variance (standard error of Cohen’s d squared) as the weight (Table 3). Among CT Image, ECAP IPG Effect, Polarity Effect, Electrode Discrimination, Low-rate Threshold, Modulation Detection Threshold, Partial Tripolar Threshold, and Multi-Dimensional Pitch Scaling, three ENI measures demonstrated significantly better speech in noise performance. Site selection intervention informed by the low-rate threshold had an effect size that was 1.66 above average (t = 3.34, *p* = 0.016, 97.5% Confidence Interval: 0.44–2.87). Similarly, modulation detection threshold had an effect size that was 1.37 higher than average (t = 2.89, *p* = 0.028, 97.5% Confidence Interval: 0.21–2.54). Meanwhile, electrode discrimination yielded an effect size that was 0.97 better (t = 3.11, *p* = 0.021, 97.5% Confidence Interval: 0.21–1.73).

**Table 3:** Weighted least square regression assessing the more suitable eni measures for site selection intervention.

ENI Measure	Coefficient	Standard error	t-statistic	*p* value	2.5% CI	97.5% CI
Low-rate threshold	1.66	0.5	3.34	0.016	0.44	2.87
Electrode discrimination	0.97	0.31	3.11	0.021	0.21	1.73
Modulation detection threshold	1.37	0.48	2.89	0.028	0.21	2.54
Partial tripolar threshold	0.33	0.59	0.56	0.597	− 1.12	1.78
ECAP IPG effect	0.07	0.42	0.16	0.878	− 0.95	1.08
Multidimensional Pitch scaling	− 2.03	1.25	− 1.62	0.157	− 5.1	1.04
Polarity effect	− 0.42	0.63	− 0.66	0.534	− 1.96	1.13

Tabe 3 Shows the result of the weighted least square regression of site selection studies (meta-regression while the weight is the inverse of the SE of the Cohen’s d) using the ENI measure as the categorical independent variable and the effect size as the dependent variable. The model adopted was “Effect Size ~ (ENI Measure—1)” to compare each category with the mean (omitting intercept). CI = Confidence Interval. Bolded *p* values indicate statistical significance.

#### Performance of the FTPM reduction interventions

**Fig. 9:**
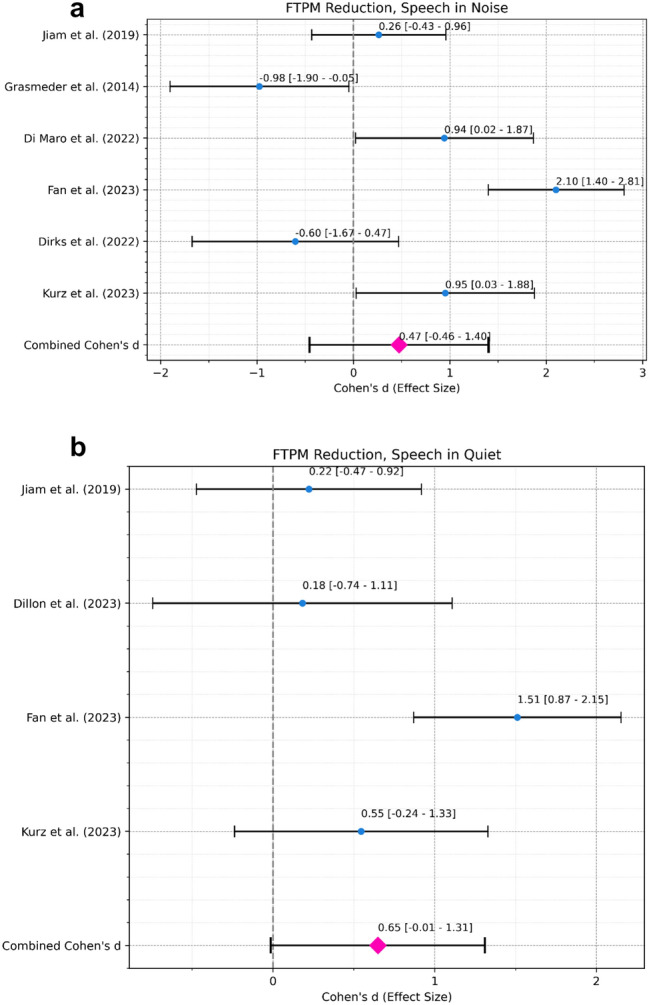
(**a**) Forest Plot for Interventions Adjusted the Frequency Allocation, Speech in Noise. Black error bars mark the 95% confidence interval. Blue dots mark the individual Cohen’s d. The pink diamond marks the combined Cohen’s d. (**b**) Forest Plot for Interventions Adjusted the Frequency Allocation, Speech in Quiet. Black error bars mark the 95% confidence interval. Blue dots mark the individual Cohen’s d. The pink diamond marks the combined Cohen’s d.

*Speech in Noise Performance*. A meta-analysis was conducted to assess the overall effect of reducing FTPM informed by ENI measures on speech in noise performance (Fig. 9a). The combined effect size, from 6 studies with 77 ears in total, was 0.47 with a 95% confidence interval ranging from − 0.46 to 1.4, indicating a moderate effect size that was not statistically significant (p = 0.32) effect. Heterogeneity was significant (Cochran’s Q = 35.33, df = 5, *p* < 0.001) indicating large differences in effect size across studies.

*Speech in Quiet Performance*. A meta-analysis was conducted to assess the overall effect of reducing FTPM informed by ENI measures on speech in quiet performance (Fig. 9b). The combined effect size, from 4 studies with 62 ears in total, was 0.65 with a 95% confidence interval ranging from − 0.01 to 1.31. Similarly, the combined effect size was large yet with a wide confidence interval indicating large but statistically near significant (*p* = 0.055) effect.

## Discussion

Re-mapping interventions informed by the ENI viability, despite being generally effective, resulted in a wide variety of outcomes. There is no clear and convincing pattern to indicate which interventions are most successful. Here we discuss the explanations of the reported findings and the potential interplay between factors.

### ENI measures suitable for site selection interventions

Electrode deactivation informed by measures intended to estimate neural health such as polarity effects & ECAP IPG measures are less successful (Table 3) than methods intended to estimate spread of excitation such as low-rate threshold, modulation detection threshold, and electrode discrimination. A possible explanation for this difference is that deactivating an electrode with a good neural survival and large spread of excitation can reduce channel interactions at the cost of not stimulating the healthy SGCs at low current level. By contrast, if the situation reverses, deactivating a site with poor neural health and small spread of excitation, there is no room of improvement in terms of channel interactions. For sites with poor neural health, increasing the T-level could recruit the neighbouring SGCs, which could result in a more discriminable stimulation pattern. Zhou and Pfingst^119^ increased the T-level at sites with a poor MDT and indeed reported improved performance.

Imaging tools were proposed to inform the site selection. However, the effect size did not differ significantly from the average of all site selection interventions (Table 3). On the other hand, the best three ENI measures for site selection interventions, low-rate threshold, modulation detection threshold, and electrode discrimination, were all behavioural tasks that focus on an important aspect of CI hearing. Electrode discrimination tests the channel independence. Modulation detection forms the backbone of CI hearing since the envelope information is coded as the modulation of a pulse train.

Some challenges arise when deactivating channels. If deactivation results in an increase in pulse rate this could influence performance and should be checked, usually pulse width can be adjusted to avoid a pulse rate increase while holding the current level and loudness similar. Following deactivation, default behaviour of the clinical software often splits the frequency band to adjacent electrodes. This setting was used across most included deactivation studies. Other possible approaches are dropping the band (leaving a spectral hole), assigning to the basal electrode, or assigning to the apical electrode. It was reported that the four approaches did not differ significantly, but the location and number of holes are important to speech understanding^120^. Simulations also suggested that the warped frequency representation can be adapted to after trainings^121^. Further work is required to probe the mechanisms behind the observed interactions between ENI measures and the most suitable mapping rationale.

### Effect of reducing FTPM on speech perception

The effect of reducing FTPM was less robust when tested acutely and demonstrated a wide range of performance (Fig. 9a). The main source of positive effect size originated from a randomised control trial^52^ with tonal language speakers. This contrast between studies might indicate that the benefit of reduced FTPM is only perceivable after a long adaptation period and/or such benefit is more salient for the tonal language speakers. In addition, the insertion depth and electrode design could also play vital roles. One study, with Advanced Bionics CI users, was not included in the meta-analysis despite the low risk of bias due to lacking ways of computing a standardised mean difference from the published information^55^. The study reported significantly worse speech in noise performance while re-mapping to reduce FTPM. Further research is needed to clarify the interplays between the reduced FTPM and the improvements in speech perception. Another challenge presenting with this approach is that, since the electrode array doesn’t always cover the whole cochlea, reducing FTPM often involves truncating certain degree of the lower frequencies.

### Statistical power

In this review, many interventions significantly improved performance for a subset of individual’s speech in noise perception without being effective at group level. This could be partly explained by studies being underpowered. For a paired t-test, to reach a statistical power of 0.8 at 0.05 significance level, two-tail, and assuming an effect size of 0.5, 33 participants are needed (computed in Python version 3.10.12 using Statsmodels version 0.14.0^122^;). Very few articles had a sample size larger than 33. In addition, the mean sample size for the articles reviewed here is 15 and hence a large effect size of 0.7 or more is required to reach significance. As a result, among the included articles, many false negatives could be expected due to the experiments being underpowered.

### Confounding factors in CI studies

Different coding strategies could interact with the interventions differently. For instance, electrode deactivation informed by electrode discrimination showed improvement at group level^58^. However, the effect disappeared when the users were all ACE users^59^. As ACE is only stimulating N electrodes within a cycle, deactivating electrodes could have a smaller influence, than when all channels are active. Individual differences such as cognitive function, listening experience, hearing loss etiology, and other demographics could also confound with the performance. Changes to CI mapping may improve one aspect of sound delivery but could be detrimental to other aspects (e.g., detection of sound could be improved but the frequency allocations could distort speech cues). Adjustment of frequency allocation could introduce different degrees of frequency compression or expansion at different sites along the array which could distort speech cues. The adaptation period after introducing a new map could also affect the outcome. Many experiments were conducted under an acute setting, which might not show the full potential of an intervention.

Using randomised controlled trials with good sample sizes could help disentangle the confounding factors. Alternatively, cross-over designs while carefully controlling the order of experimental maps could serve as a viable option if RCT is not feasible. Overall, the same intervention could have very different outcomes across individuals and hence it is important to consider individual factors while generalising the experimental findings to the clinical practice.

### Limitations of the review

Initial screening of the articles was conducted by a single reviewer following very clear criteria. The reviewer took caution to avoid false exclusion by making use of abstract and full-text level. The screened list of articles was crossed checked against reference lists to ensure that key articles were not omitted, and the two additional reviewers also checked the reference lists to ensure that relevant articles were captured. Many of the specific interventions were from one lab group so replication of findings would be beneficial. We found unexplained missing data or incomplete statistical reports in approximately one quarter of the publications.

## Conclusion

Re-mapping interventions informed by ENI measures yielded a moderate and significant effect size of 0.48 for improvements in speech in noise perception. Reducing FTPM by matching the frequency allocation to the estimated characteristic frequencies informed by imaging tools showed a similar effect size of 0.47 but was not statistically significant due to the large variability across studies reflected by a wide confidence interval covering zero effect size. Site selection interventions yielded a moderate and significant (*p* = 0.005) effect size of 0.59 on speech in noise performance. Site selection interventions were more successful when informed by the low-rate threshold, modulation detection threshold, and electrode discrimination, yielding large effect sizes around 1–1.5. Replications of studies are much needed since the same ENI measure is often reported by the same research group. Further work is required to customise the approaches to meet the needs of individual CI recipients. Overall, the viability of ENI can serve as a great information source for guiding mapping interventions.

## Electronic supplementary material

Below is the link to the electronic supplementary material.

Supplementary Material 1

Supplementary Material 2

Supplementary Material 3

Supplementary Material 4

Supplementary Material 5

Supplementary Material 6

Supplementary Material 7

## Data Availability

Data is provided within the manuscript and supplementary information files. Please contact the corresponding author if the reader is interested in anything that is not already provided.
