# Supplementary material for: Cochlear implant re-mapping informed by measures of viability of the electrode-neural interface: a systematic review with meta-analysis
Source: Sci Rep. 2025 Jul 30;15:27795. doi: 10.1038/s41598-025-09610-x (PMC12310961; doi:10.1038/s41598-025-09610-x)
Supplement: Supplementary file 2 — Supplementary Material 2 [file 41598_2025_9610_MOESM2_ESM.docx]

**Appendices**

**Appendix A: Search Terms for Databases**

Please check the corresponding PDF file for each database.

**Appendix B: Risk of Bias Questionnaire**

Risk of bias assessment questionnaire

for cochlear implant intervention studies

## **I. Background**

Several tools are available for assessing the quality of a study (CEBM, CASP, Cochrane); however, the tools apply different questions to different study designs. Consequently, the direct comparison between studies differing in designs is hard to achieve with conventional tools. In addition, interventions based on changes in mapping of cochlear implants are relatively reversible compared with surgical or pharmaceutical interventions, which blurs the distinction between a RCT and a cohort-like study if the biases are well-considered by the researcher.

This questionnaire is designed to identify the risk of bias in several aspects compared with the ideal way of conducting the study, referred to as the target study in the following. Nine domains that might affect the validity of the study are assessed. For each domain, several screening questions are proposed to help the assessor judge the level of risk. After screening questions, assessors should consider the impact of this bias compared with the best feasible way of addressing the bias.

## **II. Markings**

The assessed study:

The study which is rated by assessors using this questionnaire.

The target study:

For each assessed study, without altering the study type (e.g., case-control to RCT), think of the best way (including design, conduction, analysis, reporting, and concluding) of answering the research question. And this best way is referred as the target study; it could be unethical or resource-consuming, but it must be feasible. (For instance, in terms of statistical power, the more participants included, the stronger power yielded. Yet, a participant number of 7 billion without a good justification such as power calculation is considered unfeasible.) As a result, it should have the lowest possible bias in each assessed domain while remaining practical.

Answering the questions satisfactorily:

When the assessed study addresses the questioned risk of bias better then or comparably to the target study, the question is answered satisfactorily. If the questions is not applicable for the assessed study, skip the question.

Domain Marking Criteria:

| Risk | Criteria |
| --- | --- |
| Low | The assessed study answers all questions satisfactorily.  AND  The bias has a low chance of invaliding results. |
| Moderate | The assessed study answers two third of the questions satisfactorily  AND  (The bias is not likely to change the statistical result or method. OR The direction of bias is likely to be towards the comparator*.) |
| High | The assessed study fails to answer two third of the questions satisfactorily.  AND  (The bias is likely to change the statistical result or method. OR The direction of bias is likely to be towards the intervention*.) |

* The aim of this tool is to identify helpful interventions in a conservative way and hence if the bias is in favour of the comparator (i.e., control or the clinical map) it’s considered relatively subtle.

Global Marking Criteria:

1 High at any domain 🡪 High

8 Lows without any High or 9 Lows 🡪 Low

7 or less Lows without any High 🡪 Moderate

Interpretation:

A cohort-like study with a low level of risk should be comparable to a RCT since they addressed the biases differently, yet, with similar efficacy.

## **III. The Questionnaire**

1. Risk of bias in study design

a. Did the study address a clearly focused issue? (i.e., with clearly defined population, intervention, comparator, and suitable outcome measures) (CASP)

b. Did the execution introduce additional factors which could affect the result? (E.g., did the authors address all the confounding factors? Could you think of other confounding factors?) (ROBINS-I / CASP)

c. How generalisable is the study? (Think about design of the implants, restrictions from the method, and could the results be generalised to most cochlear implant users. Did the participants have the same implants or a special mapping method?)

2. Risk of bias in participant selection

a. Were the recruited participants a close representation of the population which authors intended to study? (CASP)

b. Were the groupings clearly defined, pre-specified, and followed? (Did authors manipulate results by changing the grouping method?)

c. Were any participants excluded from the study after measurements conducted? If so, was the reason justified?

d. Was the reason for missing data reported?

e. Were any participants removed from the study due to missing data? (ROBINS-I)

f. Were the proportion of missing data among participants similar across groups? (ROBINS-I)

3. Risk of bias in control group

a. For randomised controlled trials, was the randomisation done without human factors?

b. Were the conditions equivalent?

c. Were the participants treated the same across groups, apart from the intervention?

d. Were the participants blinded to the treatments received?

4. Risk of bias in learning effect

a. Was the test sequence balanced (E.g., A -> B -> Comparator -> Comparator -> B -> A or measure the comparator after interventions) to tackle the learning effect? (Consider if getting more familiar with the tests could affect the results.)

b. If there is a control group without an intervention, did they undergo the same assessments?

c. If the performance with the clinical map at the end of study differs from the performance at the start of the study? (Could the results be biased due to being more experienced with the implants?)

5. Risk of bias in adaptation to the new map

a. How long did the participants have to adapt to the intervention (experimental map)?

b. In what format was the adaption implemented? (acute/chronic? live voice/recorded speech? With visual cues/written scripts?)

6. Risk of bias in outcome measure

a. Were assessors blinded to the intervention?

b. Was the measure validated and cited?

c. Were the methods of outcome assessment comparable across groups? (ROBINS-I)

7. Risk of bias in statistical inaccuracy

a. Was a power calculation conducted?

b. Was the appropriate statistical method applied? (E.g., correctly justified the use of the parametric or non-parametric method, if the sample size is adequate for the adopted statistical test)

c. Was the statistical result reported in full?

d. Was the effect size reported?

e. Was the researcher conducted the analysis blinded to the data set?

8. Risk of bias in conflict of interest

a. Was the conflict of interest disclosed?

b. Could the funder commercially benefit from the study result? If yes, was the reported result in favour of the funder?

c. Were any of the authors affiliated to the company?

d. Was it a company paper?

9. Risk of bias in reporting

a. Was the conclusion closely supported by the statistical results? (Think about over interpreting.)

b. Was the study protocol registered online and accessible? (Try to find any sign of selective reporting. For instance, a conducted but not reported outcome measurement could be a red flag.)

c. If the items of outcome measurements reported similarly across groups? (E.g., 3 measures were applied on two groups; however, only 2 measures were reported for one group while all 3 were
