# Supplementary material for: Cochlear implant re-mapping informed by measures of viability of the electrode-neural interface: a systematic review with meta-analysis
Source: Sci Rep. 2025 Jul 30;15:27795. doi: 10.1038/s41598-025-09610-x (PMC12310961; doi:10.1038/s41598-025-09610-x)
Supplement: Supplementary file 3 — Supplementary Material 3 [file 41598_2025_9610_MOESM3_ESM.pdf]

Edit Search

Search Name: Final Embase and Medline 3rd Jan 2022

Comment:

Save Cancel

Set	Search Statement	Annotations	Insert	Edit	Delete
1.	"cochlea* implant*".ti,ab.				
2.	"electrode-neural interface".ti,ab.				
3.	"spectral resolution ".ti,ab.				
4.	"channel interaction".ti,ab.				
5.	"current spread".ti,ab.				
6.	"current steering".ti,ab.				
7.	"current focusing".ti,ab.				
8.	"imag* guide*".ti,ab.				
9.	"virtual channel".ti,ab.				
10.	"phantom channel".ti,ab.				
11.	tripolar.ti,ab.				
12.	"re-mapping".ti,ab.				
13.	"remapping".ti,ab.				
14.	"modulation discrimination".ti,ab.				
15.	"frequency differen* limen*".ti,ab.				
16.	"pitch discrimination".ti,ab.				
17.	"frequency discrimination".ti,ab.				
18.	"modulation detection".ti,ab.				
19.	"Channel selection".ti,ab.				
20.	"electrode selection".ti,ab.				
21.	"deactivat*".ti,ab.				
22.	electrode.ti,ab.				
23.	channel.ti,ab.				
24.	22 or 23				
25.	21 and 24				
26.	"electrode discrimination".ti,ab.				
27.	"channel discrimination".ti,ab.				
28.	" electrically evoked compound action potential".ti,ab.				
29.	"ECAP".ti,ab.				
30.	"NRT".ti,ab.				
31.	"neural response telemetry".ti,ab.				
32.	"frequency allocation table".ti,ab.				
33.	"transimpedance matrix".ti,ab.				
34.	"temporal modulation sensitivity".ti,ab.				
35.	"patient specific".ti,ab.				
36.	"custom*".ti,ab.				
37.	"programming".ti,ab.				
38.	"mapping".ti,ab.				
39.	"stimulation site".ti,ab.				
40.	"speech".ti,ab.				
41.	"spectro temporal".ti,ab.				
42.	40 or 41				
43.	2 or 3 or 4 or 5 or 6 or 7 or 8 or 9 or 10 or 11 or 12 or 13 or 14 or 15 or 16 or 17 or 18 or 19 or 20 or 25 or 26 or 27 or 28 or 29 or 30 or 31 or 32 or 33 or 34 or 35 or 36 or 37 or 38 or 39				

44. 1 and 42 and 43
45. limit 44 to (english language and humans)
46. remove duplicates from 45
47. "spectr* ripple".ti,ab.
48. "spectr* temp*".ti,ab.
49. 40 or 47 or 48
50. 1 and 43 and 49
51. limit 50 to english language
52. limit 51 to human
53. remove duplicates from 52

Save Cancel

© 2023 Ovid Technologies, Inc. All rights reserved.

OvidUI_04.27.02.002, SourceID 33c25652d55117f5c1fe09c53f5eac7dee4621de
