# Supplementary material for: Cochlear implant re-mapping informed by measures of viability of the electrode-neural interface: a systematic review with meta-analysis
Source: Sci Rep. 2025 Jul 30;15:27795. doi: 10.1038/s41598-025-09610-x (PMC12310961; doi:10.1038/s41598-025-09610-x)
Supplement: Supplementary file 6 — Supplementary Material 6 [file 41598_2025_9610_MOESM6_ESM.pdf]

((TITLE-ABS ("spectr* ripple")) OR (TITLE-ABS ("spectr* temp*")) OR (TITLE-ABS (speech))) AND (((TITLE-ABS ("stimulation site")) OR (TITLE-ABS ("mapping")) OR (TITLE-ABS (programming)) OR (TITLE-ABS ("custom*")) OR (TITLE-ABS ("patient specific")) OR (TITLE-ABS ("temporal modulation sensitivity")) OR (TITLE-ABS ("trans*impedance matr*")) OR (TITLE-ABS ("frequency allocation table")) OR (TITLE-ABS ("neural response telemetry")) OR (TITLE-ABS (nrt)) OR (TITLE-ABS (ecap)) OR (TITLE-ABS ("electric* evoked compound action potential")) OR (TITLE-ABS ("channel discrimination")) OR (TITLE-ABS ("electrode discrimination")) OR (TITLE-ABS ("channel deactivat*")) OR (TITLE-ABS ("electrode deactivat*"))) OR ((TITLE-

ABS ("electrode selection")) OR (TITLE-ABS ("Channel selection")) OR (TITLE-ABS ("modulation detection")) OR (TITLE-ABS ("frequency discrimination")) OR (TITLE-ABS ("pitch discrimination")) OR (TITLE-ABS ("frequency differen* limen*")) OR (TITLE-ABS ("modulation discrimination")) OR (TITLE-ABS (r*mapping)) OR (TITLE-ABS (tripolar)) OR (TITLE-ABS ("phantom channel")) OR (TITLE-ABS ("virtual channel")) OR (TITLE-ABS ("imag* guide*")) OR (TITLE-ABS ("current focusing")) OR (TITLE-ABS ("current steering")) OR (TITLE-ABS ("current spread")) OR (TITLE-ABS ("channel interaction")) OR (TITLE-ABS ("spectral resolution")) OR (TITLE-ABS ("electro* neur* interface"))))) AND (TITLE-ABS ("cochlea* implant*")) AND (EXCLUDE (DOCTYPE , "re")

OR EXCLUDE (DOCTYPE , "ch") OR EXCLUDE (DOCTYPE , "cr")) AND (LIMIT-TO (LANGUAGE , "English"))
