# Supplementary material for: Cochlear implant re-mapping informed by measures of viability of the electrode-neural interface: a systematic review with meta-analysis
Source: Sci Rep. 2025 Jul 30;15:27795. doi: 10.1038/s41598-025-09610-x (PMC12310961; doi:10.1038/s41598-025-09610-x)
Supplement: Supplementary file 7 — Supplementary Material 7 [file 41598_2025_9610_MOESM7_ESM.pdf]

English Products

Web of Science™ Search Marked List History Alerts Jason Tzu-Hsien Lien

Search History

Interested in more search options?
Manage or rerun your saved searches on the Alerts page. To combine searches, go to Advanced Search.

Clear History

Type	Search Query and Results	Database	Results	Actions
**Current session**				
Search	**#4 AND #43 AND #44** and **Review Articles** (Exclude – Document Types) and **Book Chapters** (Exclude – Document Types) and **English** (Languages)<br>12:25 AM	Web of Science Core Collection<br>Show editions	1,155	
Search	**#4 AND #43 AND #44** and **Review Articles** (Exclude – Document Types) and **Book Chapters** (Exclude – Document Types) and **English** (Languages) and **Adult** (Search within all fields)	Web of Science Core Collection<br>Show editions	216	

32

12:24 AM

Search	**#4 AND #43 AND #44** and **Review Articles** (Exclude – Document Types) and **Book Chapters** (Exclude – Document Types) and **English** (Languages)<br>12:24 AM	Web of Science Core Collection<br>Show editions	1,155
Search	**#4 AND #43 AND #44** and **Review Articles** (Exclude – Document Types) and **Book Chapters** (Exclude – Document Types) and **English** (Languages) and **Human Study** (Search within all fields)<br>12:24 AM	Web of Science Core Collection<br>Show editions	383
Search	**#4 AND #43 AND #44** and **Review Articles** (Exclude – Document Types) and **Book Chapters** (Exclude – Document Types) and **English** (Languages)<br>12:23 AM	Web of Science Core Collection<br>Show editions	1,155
Search	**#4 AND #43 AND #44** and **Review Articles** (Exclude – Document Types) and **Book Chapters** (Exclude – Document Types)<br>12:22 AM	Web of Science Core Collection<br>Show editions	1,174
Search	**#4 AND #43 AND #44** and **Review Articles** (Exclude – Document Types)	Web of Science Core Collection<br>Show editions	1,182

12:21 AM

Search	**#4 AND #43 AND #44** and **English** (Languages)<br>12:14 AM	Web of Science Core Collection<br>Show editions	1,211
Search	**#4 AND #43 AND #44** and **Review Articles** or **Book Chapters** (Exclude – Document Types) and **English** (Languages)<br>12:09 AM	Web of Science Core Collection<br>Show editions	1,155
Search	**#4 AND #43 AND #44** and **Review Articles** or **Book Chapters** (Exclude – Document Types)<br>12:08 AM	Web of Science Core Collection<br>Show editions	1,174
Search	**#4 AND #43 AND #44**<br>12:05 AM	Web of Science Core Collection<br>Show editions	1,230
Search	**TS = ("cochlea* implant*")**<br>12:05 AM	Web of Science Core Collection<br>Show editions	18,079
Search	**#5 OR #6 OR #7 OR #8 OR #9 OR #10 OR #11 OR #12 OR #13 OR**	Web of Science Core	1,285,777

	#14 OR #15 OR #16 OR #17 OR #18 OR #23 OR #24 OR #25 OR #26 OR #27 OR #28 OR #29 OR #30 OR #31 OR #32 OR #33 OR #34 OR #35 OR #36 OR #37 OR #38 OR #39 OR #40 OR #41 OR #42<br>12:05 AM	Collection<br>Show editions	
Search	TS = ("electro* neur* interface")<br>12:05 AM	Web of Science Core Collection<br>Show editions	82
Search	TS = ("spectral resolution")<br>12:05 AM	Web of Science Core Collection<br>Show editions	17,928
Search	TS = ("channel interaction")<br>12:05 AM	Web of Science Core Collection<br>Show editions	670
Search	TS = ("current spread")<br>12:05 AM	Web of Science Core Collection<br>Show editions	462
Search	TS = ("current steering")<br>12:05 AM	Web of Science Core Collection<br>Show editions	1,127

Search	TS = ("current focusing")<br>12:05 AM	Web of Science Core Collection<br>Show editions	82
Search	TS = ("imag* guide*")<br>12:05 AM	Web of Science Core Collection<br>Show editions	27,294
Search	TS = ("virtual channel")<br>12:05 AM	Web of Science Core Collection<br>Show editions	913
Search	TS = ("phantom channel")<br>12:05 AM	Web of Science Core Collection<br>Show editions	6
Search	TS = ("tripolar")<br>12:05 AM	Web of Science Core Collection<br>Show editions	1,061
Search	TS = ("re-mapping")<br>12:05 AM	Web of Science Core Collection<br>Show editions	347
Search	TS = ("remapping")	Web of Science Core	3,157

	12:05 AM	Collection<br>Show editions	
Search	TS = ("modulation discrimination")<br>12:05 AM	Web of Science Core Collection<br>Show editions	26
Search	TS = ("frequency differen* limen*")<br>12:05 AM	Web of Science Core Collection<br>Show editions	134
Search	TS = ("pitch discrimination")<br>12:05 AM	Web of Science Core Collection<br>Show editions	718
Search	TS = ("frequency discrimination")<br>12:05 AM	Web of Science Core Collection<br>Show editions	2,073
Search	TS = ("modulation detection")<br>12:05 AM	Web of Science Core Collection<br>Show editions	763
Search	TS = ("Channel selection")<br>12:05 AM	Web of Science Core Collection<br>Show editions	3,056

Search	TS = ("electrode selection") 12:05 AM	Web of Science Core Collection Show editions	170
Search	#19 AND #22 12:05 AM	Web of Science Core Collection Show editions	4,178
Search	#20 OR #21 12:05 AM	Web of Science Core Collection Show editions	1,360,919
Search	TS = ("electrode") 12:05 AM	Web of Science Core Collection Show editions	473,225
Search	TS = (" channel") 12:05 AM	Web of Science Core Collection Show editions	901,159
Search	TS = ("deactivat*") 12:05 AM	Web of Science Core Collection Show editions	60,690

Search	TS = ("electrode discrimination")<br>12:05 AM	Web of Science Core Collection<br>Show editions	88	
Search	TS = ("channel discrimination")<br>12:05 AM	Web of Science Core Collection<br>Show editions	104	
Search	TS = ("electric* evoked compound action potential")<br>12:05 AM	Web of Science Core Collection<br>Show editions	230	
Search	TS = ("ECAP")<br>12:05 AM	Web of Science Core Collection<br>Show editions	5,284	
Search	TS = ("NRT")<br>12:05 AM	Web of Science Core Collection<br>Show editions	3,180	
Search	TS = ("neural response telemetry")<br>12:05 AM	Web of Science Core Collection<br>Show editions	230	
Search	TS = ("frequency allocation table")	Web of Science Core	8	

	12:05 AM	Collection<br>Show editions		
Search	TS = ("transimpedance matrix")<br>12:05 AM	Web of Science Core Collection<br>Show editions	8	
Search	TS = ("temporal modulation sensitivity")<br>12:05 AM	Web of Science Core Collection<br>Show editions	45	
Search	TS = ("patient specific")<br>12:05 AM	Web of Science Core Collection<br>Show editions	27,350	
Search	TS = ("custom*")<br>12:05 AM	Web of Science Core Collection<br>Show editions	365,666	
Search	TS = ("programming")<br>12:05 AM	Web of Science Core Collection<br>Show editions	345,644	
Search	TS = ("mapping")<br>12:05 AM	Web of Science Core Collection<br>Show editions	501,585	

Search	TS = ("stimulation site")<br>12:05 AM	Web of Science Core Collection<br>Show editions	1,132	
Search	#1 OR #2 OR #3<br>12:05 AM	Web of Science Core Collection<br>Show editions	244,482	
Search	TS = ("speech")<br>12:05 AM	Web of Science Core Collection<br>Show editions	240,752	
Search	TS = ("spectr* temp*")<br>12:05 AM	Web of Science Core Collection<br>Show editions	4,351	
Search	TS = ("spectr* ripple")<br>12:05 AM	Web of Science Core Collection<br>Show editions	167	

Tuesday, January 4

Search	#44 AND #40 AND #1	Web of Science Core Collection	1,230

32

Show editions

12:01 AM

Search	#43 OR #42 OR #41<br>12:01 AM	Web of Science Core Collection<br>Show editions	242,381
Search	TS = ("spectr* ripple")<br>12:01 AM	Web of Science Core Collection<br>Show editions	167
Search	TS = ("spectr* temp*")<br>12:00 AM	Web of Science Core Collection<br>Show editions	4,351
Search	TS = ("speech")<br>12:00 AM	Web of Science Core Collection<br>Show editions	238,651
Search	#39 OR #38 OR #37 OR #36 OR #35 OR #34 OR #33 OR #32 OR #31 OR #30 OR #29 OR #28 OR #27 OR #26 OR #25 OR #20 OR #19 OR #18 OR #17 OR #16 OR #15 OR #14 OR #13 OR #12 OR #11 OR #10 OR #9 OR #8 OR #7 OR #6 OR #5 OR #4 OR #3 OR #2<br>11:59 PM	Web of Science Core Collection<br>Show editions	1,284,607

Search	TS = ("stimulation site")<br>11:59 PM	Web of Science Core Collection<br>Show editions	1,132	
Search	TS = ("mapping")<br>11:58 PM	Web of Science Core Collection<br>Show editions	501,476	
Search	TS = ("programming")<br>11:58 PM	Web of Science Core Collection<br>Show editions	345,621	
Search	TS = ("custom*")<br>11:58 PM	Web of Science Core Collection<br>Show editions	364,634	
Search	TS = ("patient specific")<br>11:58 PM	Web of Science Core Collection<br>Show editions	27,350	
Search	TS = ("temporal modulation sensitivity")<br>11:58 PM	Web of Science Core Collection<br>Show editions	45	
Search	TS = ("transimpedance matrix")	Web of Science Core Collection<br>Show editions	8	32

11:57 PM

Search	TS = ("frequency allocation table")<br>11:57 PM	Web of Science Core Collection<br>Show editions	8	
Search	TS = ("neural response telemetry")<br>11:57 PM	Web of Science Core Collection<br>Show editions	230	
Search	TS = ("NRT")<br>11:56 PM	Web of Science Core Collection<br>Show editions	3,180	
Search	TS = ("ECAP")<br>11:56 PM	Web of Science Core Collection<br>Show editions	5,284	
Search	TS = ("electric* evoked compound action potential")<br>11:56 PM	Web of Science Core Collection<br>Show editions	230	
Search	TS = ("channel discrimination")<br>11:55 PM	Web of Science Core Collection<br>Show editions	104	

Search	TS = ("electrode discrimination")<br>11:55 PM	Web of Science Core Collection<br>Show editions	88	
Search	#21 AND #24<br>11:55 PM	Web of Science Core Collection<br>Show editions	4,178	
Search	#23 OR #22<br>11:54 PM	Web of Science Core Collection<br>Show editions	1,360,836	
Search	TS = (" channel")<br>11:54 PM	Web of Science Core Collection<br>Show editions	901,077	
Search	TS = ("electrode")<br>11:53 PM	Web of Science Core Collection<br>Show editions	473,224	
Search	TS = ("deactivat*")<br>11:53 PM	Web of Science Core Collection<br>Show editions	60,690	
Search	TS = ("electrode selection")	Web of Science Core Collection	170	32

	11:53 PM	Show editions		
Search	TS = ("Channel selection") 11:53 PM	Web of Science Core Collection Show editions	3,056	
Search	TS = ("modulation detection") 11:52 PM	Web of Science Core Collection Show editions	763	
Search	TS = ("frequency discrimination") 11:52 PM	Web of Science Core Collection Show editions	2,073	
Search	TS = ("pitch discrimination") 11:52 PM	Web of Science Core Collection Show editions	712	
Search	TS = ("frequency differen* limen*") 11:52 PM	Web of Science Core Collection Show editions	134	
Search	TS = ("modulation discrimination") 11:51 PM	Web of Science Core Collection Show editions	26	

Search	TS = ("remapping")<br>11:51 PM	Web of Science Core Collection<br>Show editions	3,157
Search	TS = ("re-mapping")<br>11:51 PM	Web of Science Core Collection<br>Show editions	347
Search	TS = ("tripolar")<br>11:49 PM	Web of Science Core Collection<br>Show editions	1,061
Search	TS = ("phantom channel")<br>11:49 PM	Web of Science Core Collection<br>Show editions	6
Search	TS = ("virtual channel")<br>11:49 PM	Web of Science Core Collection<br>Show editions	913
Search	TS = ("imag* guide*")<br>11:49 PM	Web of Science Core Collection<br>Show editions	27,294
Search	TS = ("current focusing")	Web of Science Core Collection	82

	11:48 PM		Show editions	
Search	**TS = ("current steering")**	11:48 PM	Web of Science Core Collection<br>Show editions	1,127
Search	**TS = ("current spread")**	11:48 PM	Web of Science Core Collection<br>Show editions	462
Search	**TS = ("channel interaction")**	11:47 PM	Web of Science Core Collection<br>Show editions	670
Search	**TS = ("spectral resolution")**	11:47 PM	Web of Science Core Collection<br>Show editions	17,928
Search	**TS = ("electro* neur* interface")**	11:46 PM	Web of Science Core Collection<br>Show editions	82
Search	**TS = ("cochlea* implant*")**	11:45 PM	Web of Science Core Collection<br>Show editions	18,079

© 2021 Clarivate
Training Portal
Product Support

Data Correction
Privacy Statement
Newsletter

Copyright Notice
Cookie Policy
Terms of Use

Manage cookies preferences

Follow Us
